# New data on *Garra makiensis* (Cyprinidae, Labeoinae) from the Awash River (Ethiopia) with remarks on its relationships to congeners on the Arabian Peninsula

**DOI:** 10.3897/zookeys.984.55982

**Published:** 2020-11-04

**Authors:** Gernot K. Englmaier, Nuria Viñuela Rodríguez, Herwig Waidbacher, Anja Palandačić, Genanaw Tesfaye, Wolfgang Gessl, Paul Meulenbroek

**Affiliations:** 1 University of Graz, Institute of Biology, Universitätsplatz 2, A-8010 Graz, Austria University of Graz Graz Austria; 2 Department of Ecology, Faculty of Science, Charles University, Viničná 7, CZ-12844 Prague 2, Czech Republic Charles University Prague Czech Republic; 3 University of Natural Resources and Life Sciences, Institute of Hydrobiology and Aquatic Ecosystem Management (IHG), Vienna, Gregor-Mendel Straße 33, A-1180 Vienna, Austria University of Natural Resources and Life Sciences Vienna Austria; 4 Natural History Museum Vienna, Burgring 7, A-1010 Vienna, Austria Natural History Museum Vienna Austria; 5 National Fisheries and Aquatic Life Research Centre, P.O. Box: 64, Sebeta, Ethiopia National Fisheries and Aquatic Life Research Centre Sebeta Ethiopia

**Keywords:** Biogeography, biodiversity, CO1 sequence data, East Africa, freshwater fish, tubercles

## Abstract

On the African continent, the genus *Garra* consists of several species often insufficiently separated from each other by diagnostic characters. Herein, a detailed morphological redescription of *Garra makiensis* from the Awash River drainage is presented, together with additional data on the type specimens of *G. makiensis* and *G. rothschildi*. Mitochondrial CO1 sequence data are also provided, including the historic paralectotype of *G. makiensis*, with a comparison to *Garra* species from Africa and the Middle East. Based on these sequences, *G. makiensis* clusters outside the group of African congeners and is a sister lineage to species from the south-east of the Arabian Peninsula. Although morphologically variable, *G. makiensis* is characterised by having a single unbranched pectoral-fin ray, a short distance between vent and anal-fin origin (7.3–19.7 % of pelvic – anal distance), chest and belly covered with scales, and a prominent axillary scale at base of pelvic fin (18.8–35.5 % of pelvic-fin length).

## Introduction

The endorheic Awash River drainage in the northern part of the Main Ethiopian Rift (MER) is subdivided into two freshwater ecoregions, the Ethiopian Highlands and the Northern Eastern Rift (Abell et al. 2008). It originates close to Ethiopia’s capital city Addis Ababa at an altitude of > 3,000 m a.s.l. Along its course (1,250 km in length), it flows from the highlands into the MER, and drains into saline Lake Abbe at the Ethiopian-Djibouti border. Numerous smaller sub-drainage systems, among them the Gotta River, belong to the Awash catchment. Biogeographically, the region is classified as part of the Abyssinian Highlands ichthyofaunal province (Roberts 1975) (a sub-province of the Nilo-Sudanic province according to Snoeks and Getahun (2013)) or the Ethiopian Rift Valley province (Paugy 2010). Evidence for ichthyofaunal affinities with the Nile River system and the Central MER were recently provided by Beshera and Harris (2014) for the *Labeobarbus intermedius* complex and by Englmaier et al. (2020a) for small-sized smiliogastrin barbs (*Enteromius* Cope, 1867).

The fish fauna of the Awash is commonly described as “impoverished” (Roberts 1975: 291) with 10–13 species belonging to five families (Golubtsov et al. 2002; Englmaier et al. 2020a, 2020b). One of the poorly investigated groups of freshwater fishes in the region is the Afro-Asian genus *Garra* Hamilton, 1822. Menon (1964) and later Getahun (2000) and Stiassny and Getahun (2007) provided the first comprehensive morphological studies on African *Garra*. Twenty-four valid species of *Garra* are currently recognised in Africa (Moritz et al. 2019). They are distributed from North Africa (Nile River in Egypt) to drainage systems in West Africa (e.g., Senegal River) and central sub-Saharan Africa (Tanzania and Angola) (Daget et al. 1984; Getahun 2000; Stiassny and Getahun 2007; Habteselassie et al. 2010; Moritz et al. 2019). With 12 species, the genus was found to be particularly diverse in the Ethiopian Highlands and surrounding drainage systems (Stiassny and Getahun 2007). In the Awash River, recent surveys gave evidence for three well supported mitochondrial clades of *Garra*, identified as: *G. aethiopica* (Pellegrin, 1927), *G. dembeensis* (Rüppell, 1835) and *G. makiensis* (Boulenger, 1903) by Englmaier et al. (2020b).

Compared to *Garra* species in Asia and the Middle East (e.g., Krupp 1983; Yang et al. 2012; Sayyadzadeh et al. 2015; Esmaeili et al. 2016; Hashemzadeh Segherloo et al. 2016; Nebeshwar and Vishwanath 2017; Kirchner et al. 2020; Kottelat 2020), systematic relationships and diagnostic morphological characters of African taxa have not been well investigated (Stiassny and Getahun 2007). So far, no consistent opinion has been reached in assessing important diagnostic characters such as the presence/absence of a red or black blotch behind the upper edge of operculum, the scale pattern on ventral side, the size and shape of the gular disc (also referred to as “mental adhesive disc” in Zhang et al. (2002)) or the tuberculation on snout and head (Menon 1964; Getahun 2000; Golubtsov et al. 2002, 2012; Stiassny and Getahun 2007). Together with recent reports of considerable intraspecific morphological variability (Golubtsov et al. 2012; Englmaier 2018), this complicates species-level determination and has led to different taxonomic opinions and inconsistent distribution records of many African species (see Getahun 2000; Golubtsov et al. 2002, 2012; Stiassny and Getahun 2007; Stiassny et al. 2007; Habteselassie 2009, 2012; Moritz et al. 2019).

One such problematic species, *G. makiensis*, was described from the Meki River (endorheic basin of Lake Ziway) in the Central MER (Boulenger 1903). In the first comprehensive revision of the genus, Menon (1964) provided a redescription of this species and included *G. rothschildi* (Pellegrin, 1905), described from the Gotta River in the Northern MER, as a synonym. This opinion was later corroborated by Getahun (2000) and Stiassny and Getahun (2007), who extended the distribution range of *G. makiensis* to the Southern MER, the Blue and White Nile, and the Omo River drainage. However, during recent surveys in the Awash River, preliminary observations showed that diagnostic characters described for *G. makiensis* in recent literature and identification keys (e.g., Stiassny and Getahun 2007; Habteselassie 2012) contain uncertainty and did not allow reliable species identification (Englmaier 2018).

Therefore, as a first step towards resolving taxonomic inconsistencies among African *Garra*, we present a detailed redescription of *G. makiensis* based on specimens from the Awash River drainage with new data on the type specimens of *G. makiensis* and *G. rothschildi*. Moreover, we provide mitochondrial CO1 sequence data for *Garra* species from the Awash River (*G. aethiopica*, *G. dembeensis*, *G. makiensis*), and the first CO1 sequence of the historic paralectotype of *G. makiensis* (BMNH 1905.7.25.88) in order to evaluate their phylogenetic relationships. These considerations are complemented with a morphological comparison of *G. makiensis* with closely related species and remarks on biogeographical implications.

## Materials and methods

**Figure 1.**
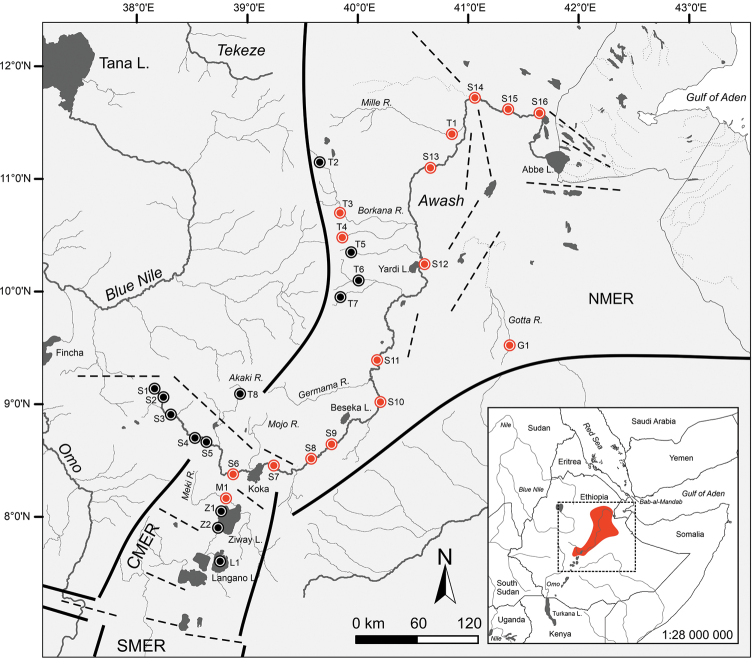
Map of the study area in the Northern and Central Main Ethiopian Rift (NMER, CMER) showing sampling sites and examined material; thick lines denoting Main Rift faults, dashed lines showing transversal faults (Bonini et al. 2005). Study sites: **S1–S6** and **T1–T8** Awash River drainage, **G1** Gotta River (type locality of *G. rothschildi*), **M1** Meki River (type locality of *G. makiensis*), **Z1–Z2** Lake Ziway, and **L1** Lake Langano. Sampling sites in red showing localities where *G. makiensis* is known from examined material or was recorded during recent surveys. The small inserted map showing known distribution range of *G. makiensis* in the Awash and Meki River drainages.

Specimens of *Garra* were collected in the Awash River, including its major tributaries (Fig. 1 and Table 1, Suppl. material 1: Table S1). Collections were made during the dry seasons between 2017 and 2019. Sampling methods are described in Englmaier et al. (2020a). After anaesthesia with etheric clove oil (*Eugenia caryophyllata*) diluted in water, fish specimens were fixed in 6 % pH neutral formalin (later stored in 75 % ethanol) or 96 % ethanol.

*Garra* species from the Awash River were identified as morphospecies based on external diagnostic characters (Englmaier et al. 2020b). In addition to comparison with type material (for *G. makiensis* and *G. aethiopica*) and original descriptions (Rüppell 1835; Boulenger 1903; Pellegrin 1927), the following available literature was used for identification: Getahun (2000), Golubtsov et al. (2002), Stiassny and Getahun (2007), Habteselassie et al. (2010), Habteselassie (2012), and Moritz et al. (2019).

Museum samples included specimens deposited in the collections of the Natural History Museum Vienna (**NMW**; Fig. 1: sampling sites S6–S16, T1, T3–T4); the British Museum of Natural History (**BMNH**; Fig. 1: sampling site M1); the American Museum of Natural History (**AMNH**; Fig. 1: sampling site G1); the Muséum national d’Histoire naturelle, Paris (**MNHN**); and the Musée royal de l’Afrique centrale, Tervuren (**MRAC**). Comparative material is listed in Table 1.

**Table 1.** Comparative material used in the present study. Sampling sites referring to those given in Fig. 1.

Taxon name	Museum number	n	Types	SL, mm	Sampling site	Information
*Discognathus makiensis*	BMNH 1905.7.25.87	1	lectotype	67.1	M1	Maki [Meki] River, Ethiopia, coll. O. Neumann and C. v. Erlanger
*Discognathus makiensis*	BMNH 1905.7.25.88	1	paralectotype	47.6	M1	Maki [Meki] River, Ethiopia, coll. O. Neumann and C. v. Erlanger (voucher specimen for CO1 (MT946130))
*Discognathus rothschildi*	MNHN 1905-0246	1	syntype	135.3	G1	Gotta [Gota] River, Ethiopia (photographs and radiographs examined)
*Discognathus rothschildi*	MNHN 1905-0247	1	syntype	108.7	G1	Gotta [Gota] River, Ethiopia (photographs and radiographs examined)
*Garra makiensis*	MRAC 91-051-P-0044	21	non-types	68.9–44.4	G1	Gota [Gotta] River, Harar province, Ethiopia (radiographs examined)
*Garra makiensis*	AMNH 227323	3	non-types	72.6–76.1	G1	Errer Gota [Gotta] River, Eastern side of Errer town, pools near main road, Hararge, Ethiopia (09°30'N, 41°15'E) (radiographs examined)
*Garra makiensis*	NMW 99222	3	non-types	44.6–136.1	S9	Awash River at Nur Sada (8°33'9"N, 39°38'10"E; 1,214 m a.s.l.), Ethiopia, 31.01.2018, coll. G.K. Englmaier, G. Tesfaye, P. Meulenbroek and H. Waidbacher (one voucher specimen for CO1 (MT946129))
*Garra makiensis*	NMW 99223	6	non-types	56.0–78.3	S7	Awash River at Wonji (8°28'23"N, 39°12'43"E; 1,552 m a.s.l.), Ethiopia, 09.11.2017, coll. G.K. Englmaier, G. Tesfaye and P. Meulenbroek (one voucher specimen for CO1 (MT946124))
*Garra makiensis*	NMW 99224	9	non-types	43.3– 90.6	S6	Awash River at Lafessa (8°23'16"N, 38°54'30"E; 1,608 m a.s.l.), Ethiopia, 08.11.2017, coll. G.K. Englmaier, G. Tesfaye and P. Meulenbroek (two voucher specimens for CO1 (MT946122, MT946123))
*Garra makiensis*	NMW 99225	3	non-types	70.6–71.4	S11	Awash River at Worer (9°20'6"N, 40°10'19"E; 743 m a.s.l.), Ethiopia, 29.01.2018, coll. G.K. Englmaier, G. Tesfaye, P. Meulenbroek and H. Waidbacher.
*Garra makiensis*	NMW 99226	1	non-types	43.4	S8	Awash River at Korkada (8°30'2"N, 39°33'7"E; 1,260 m a.s.l.), Ethiopia, 09.12.2017, coll. G.K. Englmaier and G. Tesfaye.
*Garra makiensis*	NMW 99230	5	non-types	66.8– 92.6	S12	Awash River at Kada Bada (10°13'53"N, 40°34'43"E; 570 m a.s.l.), Ethiopia, 28.01.2018, coll. G.K. Englmaier, G. Tesfaye, P. Meulenbroek and H. Waidbacher (two voucher specimens for CO1 (MT946125, MT946126))
*Garra makiensis*	NMW 99231	16	non-types	49.1–119.9	S10	Awash River at Yimre (9°4'59"N, 40°10'3"E; 797 m a.s.l.), Ethiopia, 30.01.2018, coll. G.K. Englmaier, G. Tesfaye, P. Meulenbroek and H. Waidbacher (two voucher specimens for CO1 (MT946127, MT946128))
*Garra makiensis*	NMW 99485	12	non-types	59.3– 97.0	S13	Awash River at Adayitu (11°7'48"N, 40°46'3"E; 460 m a.s.l.), Ethiopia, 12.03.2019, coll. G.K. Englmaier, G. Tesfaye, P. Meulenbroek and H. Waidbacher.
*Garra makiensis*	NMW 99489	4	non-types	53.5– 82.2	T1	Lower Mille River (11°24'50"N, 40°45'37"E; 482 m a.s.l.), Ethiopia, 12.03.2019, coll. G.K. Englmaier, G. Tesfaye, P. Meulenbroek and H. Waidbacher.
*Garra makiensis*	NMW 99491	7	non-types	42.2– 106.1	S14	Awash River at Dubti (11°41'50"N, 41°7'23"E; 378 m a.s.l.), Ethiopia, 13.03.2019, coll. G.K. Englmaier, G. Tesfaye, P. Meulenbroek and H. Waidbacher.
*Garra makiensis*	NMW 99504	2	non-types	141.3– 147.9	T4	Jara River (10°31'14"N, 39°57'13"E; 1,434 m a.s.l.), Ethiopia, 17.03.2019, coll. G.K. Englmaier, G. Tesfaye, P. Meulenbroek and H. Waidbacher.
*Garra makiensis*	NMW 99507	3	non-types	99.2– 118.2	T3	Middle Borkana River (10°38'09"N, 39°55'54"E; 1,417 m a.s.l.), Ethiopia, 17.03.2019, coll. G.K. Englmaier, G. Tesfaye, P. Meulenbroek and H. Waidbacher.

In the present study, we refer to the species names *Garra smarti* Krupp & Budd, 2009 and *Garra sindhi* Lyon, Geiger & Freyhof, 2016, although the specific epithet of both species was recently ‘corrected’ to *smartae* and *sindhae* by Kirchner et al. (2020). Krupp and Budd (2009) and Lyon et al. (2016) dedicated the species names to two different women, using the masculine genitive ending -*i*, instead of the common feminine genitive ending -*ae.* However, the names *smarti* and *sindhi* are not to be considered incorrect according to Art. 32.5 of the International Code of Zoological Nomenclature (1999) and are therefore not to be modified (see also Dubois (2007) and Nemésio and Dubois (2012) for species names derived from personal names).

### Morphological analyses

**Figure 2.**
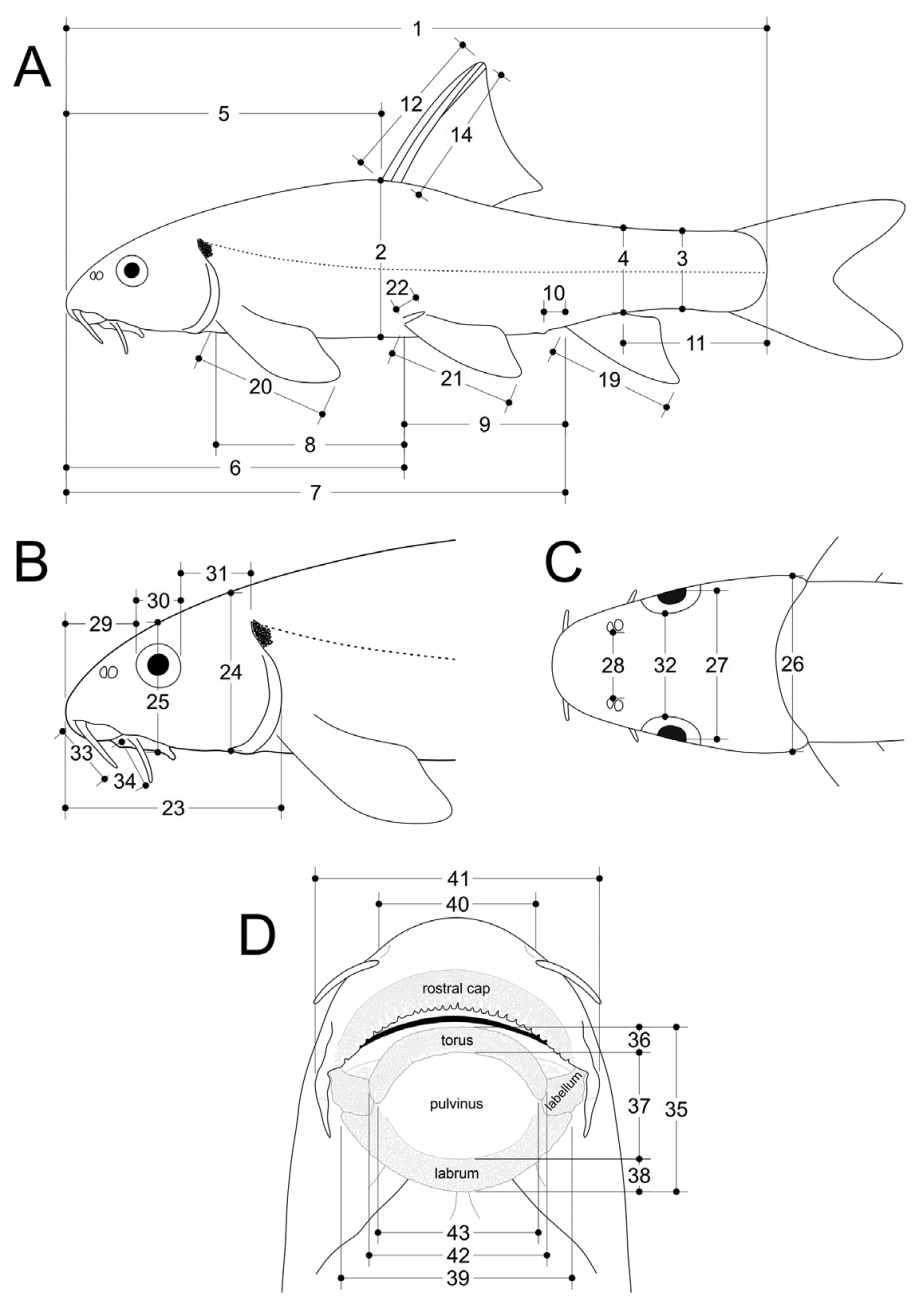
Schematic illustration of **A** body measurements **B** (lateral) and **C** (dorsal) head measurements, and **D** (ventral) head and gular disc (as defined in Kottelat (2020)) measurements. For a detailed description see Suppl. material 1: Table S2. 1, standard length (SL); 2, body depth at dorsal-fin origin; 3, minimum caudal-peduncle depth; 4, maximal caudal-peduncle depth; 5, predorsal length; 6, prepelvic length; 7, preanal length; 8, pectoral – pelvic distance; 9, pelvic – anal distance; 10, vent distance; 11, caudal-peduncle length; 12, dorsal-fin depth; 14, depth of 1^st^ branched dorsal-fin ray; 19, anal-fin depth; 20, pectoral-fin length; 21, pelvic-fin length; 22, length of axillary scale; 23, head length; 24, head depth at nape; 25, head depth at eye; 26, head width at posterior end of operculum; 27, head width at eyes; 28, width between nostrils; 29, snout length; 30, eye horizontal diameter; 31, orbit – operculum distance; 32, interorbital width; 33, anterior barbel length; 34, posterior barbel length; 35, disc length; 36, length of torus; 37, length of pulvinus; 38, length of labrum; 39, disc width; 40, width between anterior barbels; 41, width of mouth; 42, width of torus; 43, width of pulvinus. Not illustrated are: 13, depth of last unbranched dorsal-fin ray; 15, depth of 2^nd^ branched dorsal-fin ray; 16, depth of 3^rd^ branched dorsal-fin ray; 17, depth of 4^th^ branched dorsal-fin ray; 18, depth of 5^th^ branched dorsal-fin ray.

In total, 124 specimens were examined, including type specimens of *G. makiensis* and *G. rothschildi*. A maximum of 43 measurements (seven for the gular disc), 22 external body counts, and nine axial skeleton counts (from x-rays) were taken. Type specimens of *G. rothschildi* were examined from photographs and radiographs, and only meristic counts were taken. Measurements and counts are defined in Suppl. material 1: Table S2; and measurements illustrated in Fig. 2.

Most measurements follow Hubbs and Lagler (1958) and Holčík et al. (1989) and were made point to point using a digital calliper to the nearest 0.1 mm. Head length (HL) excludes the skin fold on the operculum. Length of the axillary scale was measured from the anteriormost to the posteriormost extremity. Length of the dorsal-fin rays was measured from the visible base of the ray to the end of the uppermost flexible part. We refer to the postpelvic region as an area on the ventral side between the insertion of the pelvic fins and the anterior margin of anus. Scales in the postpelvic region were counted along midline. The terminology used for the external oral and gular morphology, including the gular disc (referred to as “disc” or “mental adhesive disc” in Zhang et al. (2002) and Stiassny and Getahun (2007)) follows Kottelat (2020). Measurements of the gular disc were done as follows (Fig. 2 and Suppl. material 1: Table S2): 35, Disc length: Distance between the anteriormedian border of torus and the posteriormost point of labrum at midline. 36, Length of torus: Distance between the anterior- and posteriormedian borders at midline. 37, Length of pulvinus: Distance between the anterior and posterior extremities of pulvinus at midline. 38, Length of labrum: Distance between the anterior and posterior extremities of labrum at midline. 39, Disc width: Maximum width of labrum at intercept with labellum. 42, Width of torus: Distance between the lateral extremities of torus. 43, Width of pulvinus: Maximum width of pulvinus between lateral extremities. The terminology used for nuptial tubercles and grooves on the snout follows Nebeshwar and Vishwanath (2017) as described for *Garra*.

External meristic counts follow Skelton (1980) and those summarised in Englmaier et al. (2020a) (Suppl. material 1: Table S2). The posterior two branched rays in the dorsal and anal fins, located on the last complex proximal pterygiophore of the fin, were counted as two. As the anteriormost unbranched rays of the dorsal and anal fins are usually deeply embedded, ray counts for those fins were taken from radiographs. Total number of lateral-series scales were counted from the first scale behind the opercular opening to the last scale on the caudal fin (bearing the lateral-line canal or without the canal). Counts and terminology of the axial skeleton follow Naseka (1996). Vertebral counts and supraneural bones were examined from radiographs.

Multivariate analyses in the form of principal component analysis (PCA) and discriminant function analysis (DFA), were used to compare type specimens of *G. makiensis* (Meki River) with those species found in the adjacent Awash River. Data for *G. aethiopica* and *G. dembeensis* were taken from Englmaier (2018). Therefore, the dataset was reduced to the number of characters used in Englmaier (2018), following the number of morphometric and meristic characters introduced by Stiassny and Getahun (2007) for African *Garra*. Primary data and basic statistics are given in Suppl. material 1: Tables S3–S5. Statistical analyses were performed in Microsoft Excel and IBM SPSS Statistics v. 26.

### Molecular analyses

Methods for DNA extraction, PCR amplification (using primers Fish-Co1-F and Fish-Co1-R according to Baldwin et al. (2009)) and sequencing of freshly sampled material (2017–2018) are described in Englmaier et al. (2020a).

For DNA extraction of historic museum material (BMNH 1905.7.25.88, *G. makiensis*, paralectotype) we used tissue from the branchial arches (right side of the specimen). DNA was extracted using the QIAamp DNA Mini and Blood Mini Kit (Qiagen) following the manufacturer’s protocol. Final DNA concentration was 23.4 ng μl^-1^. All lab work was performed in a DNA clean room with sterilised and UV radiated utensils. Because museum DNA is typically fragmented, we designed specific primers to amplify approximately 150 bp long fragments of the cytochrome *c* oxidase subunit 1 (CO1) (Table 2). Primers were designed based on the CO1 alignment of the extant *Garra* samples included in this study using Primer-BLAST (NCBI), and were arranged in a way that adjacent fragments extensively overlap. PCR reactions were done in 50 μl, with 5 μl buffer, 4 μl MgCl_2_ (2.0 mM), 2 μl Enhancer, 1 μl dNTPs (500 μM), 0.50 μl of each primer (50 pmol μl^-1^, for primer sequences see Table 2), 0.4 μl of AmpliTaq Gold 360 DNA Polymerase (1 unit) and 2–3 μl of DNA. The same touch-down PCR protocol was used for all primer pairs, with initial denaturation at 95 °C for 10 min, followed by five cycles at 95 °C for 30 s, 63 °C for 2 min and 72 °C for 45 s, and 40 cycles at 95 °C for 45 s, 61 °C for 45 s and 72 °C for 45 s. Final extension was performed at 72 °C for 7 min. PCR products were purified with a Qiagen PCR purification kit, and purified PCR products were sequenced (in both directions) by Microsynth with PCR primers. After amplification, fragments were aligned with MEGA6 (Tamura et al. 2011) and composed into a single sequence (451 bp total length).

**Table 2.** Sequences (5'–3') of primers used for the PCR reactions of the historic paralectotype of *Garra makiensis* (BMNH 1905.7.25.88).

Primer	Sequence (5'–3')	Product (incl. primers)
Garra_new_4_F	GTTACTGCCCACGCTTTTGT	185 bp
Garra_new_4_R	CTTCGACTCCAGAGGAGGCT	
Garra_new_5_F	AGCCTCCTCTGGAGTCGAAG	191 bp
Garra_new_5_R	GGGAAATGGCTGGGGGTTTT	
Garra_new_6_F	GGGGTTTTGGAAACTGACTCG	195 bp
Garra_new_6_R	ATGCTCCTGCGTGAGCTAAG	
Garra_new_7_F	CTGCATCTAGCAGGGGTGTC	176 bp
Garra_new_7_R	ATCGTAATTCCGGCAGCTAGT	
Garra_new_10_F	CCAGATATGGCATTTCCACGG	155 bp
Garra_new_10_R	GCTCCTGCGTGAGCTAAGTT	

The dataset used for phylogenetic analysis comprised 13 original CO1 sequences (611 bp) from freshly collected samples of *G. aethiopica*, *G. dembeensis* and *G. makiensis*, and the historic paralectotype sequence (451 bp). Obtained sequences were deposited on GenBank under accession numbers MT946118–MT946130. Additionally, we included 46 sequences retrieved from GenBank, corresponding to different *Garra* species from Africa and the Arabian Peninsula. An Asian *Garra* species was included as outgroup following Yang et al. (2012) (Suppl. material 1: Table S6).

Sequences were edited in MEGA7 (Kumar et al. 2016) and aligned with ClustalW. PartitionFinder2 (Lanfear et al. 2017) was used to estimate the best partition scheme and best fit substitution model. Phylogenetic reconstruction was conducted using Maximum Likelihood (ML) and Bayesian Inference (BI) methods. ML was performed using RAxML-HPC2 Workflow on XSEDE (v. 8.2.12) through CIPRES Science Gateway (Miller et al. 2011), with 1,000 bootstrap replicates. For BI, MrBayes v. 3.2.6 (Ronquist et al. 2012) was used. Two independent Markov Chain Monte Carlo (MCMC) were run simultaneously for 5 million generations with sampling trees every 500 generations. The first 25 % of obtained trees was discarded as ‘burn-in’. All computed trees were visualised and edited with FigTree v. 1.4.4 (Rambaut 2012) and Inkscape v. 0.92.3. MEGA7 (Kumar et al. 2015) was used to calculate between group mean distances (uncorrected p-distances).

Two different sequence alignments were used for calculating the p-distances: the first alignment did not include the paralectotype sequence of *G. makiensis* and each sequence had a length of 611 bp; the second dataset was trimmed to the length of the paralectotype sequence (451 bp) and was used to compare the paralectotype with the remaining studied species.

## Results

**Figure 3.**
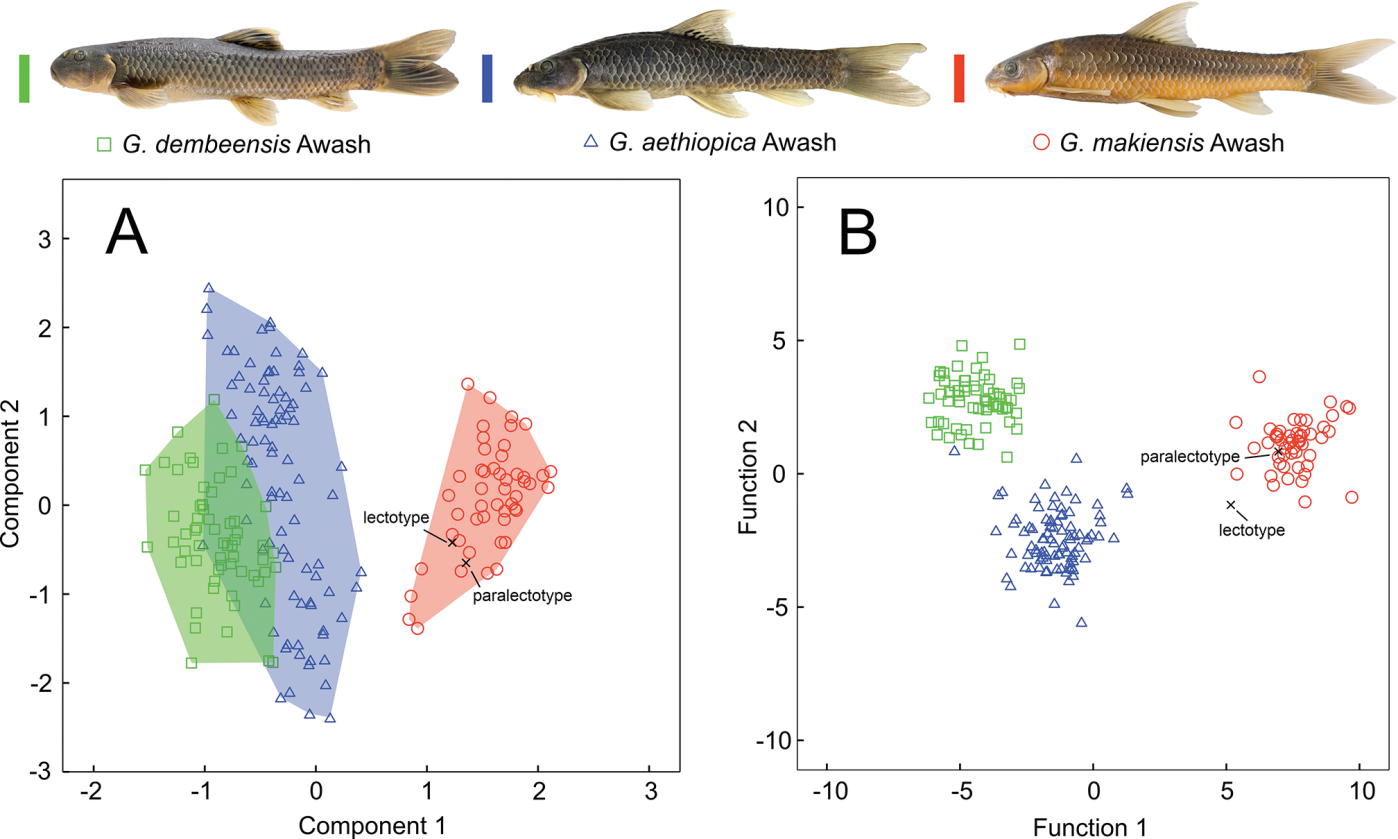
Results of **A**PCA and **B**DFA, comparing *Garra* species from the Awash River with type specimens of *G. makiensis* from the Meki River (lectotype: BMNH 1905.7.25.87, paralectotype: BMNH 1905.7.25.88). Analyses are based on 17 morphometric and six meristic characters as given in Suppl. material 1: Table S3.

Identification of the Awash samples as *G. makiensis* is based on 1) the morphological comparison to type specimens of *G. makiensis* and *G. rothschildi* (Tables 3, 4), including multivariate statistical analyses (PCA, DFA) of 17 morphometric and six meristic characters with a comparison to *G. aethiopica* and *G. dembeensis* from the Awash River (Fig. 3); and 2) CO1 sequence data with a p-distance of 0.08 % between the paralectotype and the samples of *G. makiensis* from the Awash River, and 9.53–11.31 % between the paralectotype and other African species (including *G. aethiopica* and *G. dembeensis* from the Awash).

**Table 3.** Morphometric data for examined *Garra makiensis* from the Meki River (type specimens) and the Awash River drainage. Information per specimen as in Table 1.

Character states	*G. makiensis*BMNH 1905.7.25.87 lectotype	*G. makiensis*BMNH 1905.7.25.88 paralectotype	*G. makiensis* Awash River				
			n	Min	Max	Mean	S.D.
Standard length (mm)	67.1	47.6	50	42.2	147.9	79.7	21.9
**Percent of standard length**							
Body depth at dorsal-fin origin	19.1	19.8	50	17.4	25.0	21.7	1.4
Minimum caudal-peduncle depth	11.1	11.5	50	9.1	12.6	10.7	0.7
Maximal caudal-peduncle depth	12.5	13.3	50	9.6	14.4	11.8	0.9
Predorsal length	45.7	46.9	50	43.1	49.7	45.1	1.4
Prepelvic length	51.0	53.0	50	46.2	53.1	48.8	1.1
Preanal length	74.7	75.8	50	70.7	75.0	72.6	1.0
Pectoral – pelvic distance	32.0	32.2	50	24.9	30.3	28.2	1.1
Pelvic – anal distance	25.1	23.6	50	22.8	27.5	24.9	1.1
Caudal-peduncle length	17.8	19.5	50	17.2	22.9	20.2	1.1
Dorsal-fin depth	24.4	26.9	50	25.1	29.9	27.9	1.1
Anal-fin depth	19.1	19.8	50	18.6	21.9	20.1	0.8
Pectoral-fin length	22.3	23.7	50	19.3	22.7	20.9	0.8
Pelvic-fin length	19.9	20.5	50	18.1	22.3	20.5	0.8
Head length	22.2	24.6	50	19.7	27.7	21.9	1.5
Head depth at nape	14.5	15.9	50	13.2	16.5	14.6	0.6
Head depth at eye	12.8	14.1	50	10.4	14.8	12.1	0.7
Head width at posterior end of operculum	15.0	16.3	50	13.6	17.1	14.8	0.8
Head width at eyes	12.9	13.6	50	12.3	17.3	13.7	1.1
Snout length	9.3	9.6	50	7.3	13.0	9.5	1.3
Eye horizontal diameter	4.8	5.6	50	3.8	6.1	4.6	0.5
Orbit – orperculum distance	9.6	10.1	50	6.3	8.6	7.2	0.5
Interorbital width	9.6	10.4	50	10.0	12.5	10.7	0.5
Disc length	6.4	6.8	50	4.3	9.2	5.7	0.9
Disc width	6.6	7.1	50	5.4	11.9	7.1	1.5
Width between anterior barbels	5.5	5.8	50	4.7	9.6	5.9	1.1
Width of mouth	7.8	8.0	50	6.3	13.6	8.6	1.5
**Percent of head length**							
Head depth at nape	65.2	64.6	50	58.8	74.4	66.8	3.6
Head depth at eye	57.8	57.5	50	49.5	62.7	55.4	3.2
Head width at posterior end of operculum	67.5	66.1	50	59.5	73.9	67.8	3.1
Head width at eyes	58.3	55.3	50	55.3	68.9	62.7	3.1
Width between nostrils	29.1	27.8	50	25.4	36.0	31.8	2.3
Snout length	42.1	38.9	50	34.0	52.5	43.1	4.1
Eye horizontal diameter	21.9	22.6	50	17.0	24.6	21.1	1.9
Orbit – orperculum distance	43.4	41.2	50	27.3	37.7	32.7	2.4
Interorbital width	43.1	42.3	50	44.3	53.4	49.0	2.2
Anterior barbel length	14.7	15.6	50	11.5	20.6	15.8	2.3
Posterior barbel length	18.8	21.3	50	8.6	23.3	15.5	3.7
Disc length	29.0	27.6	50	21.5	33.2	25.9	2.8
Disc width	29.6	28.9	50	25.6	44.7	32.3	5.5
Width between anterior barbels	24.7	23.5	50	21.6	36.7	27.0	3.7
Width of mouth	35.2	32.5	50	30.8	52.6	39.1	5.2
**Percent of caudal peduncle length**							
Minimum caudal-peduncle depth	62.7	58.8	50	42.2	62.4	52.9	4.8
Maximal caudal-peduncle depth	70.5	68.3	50	48.5	71.0	58.3	5.4
**Percent of eye horizontal diameter**							
Anterior barbel length	67.4	69.1	50	48.6	94.7	75.4	10.1
Posterior barbel length	85.8	94.3	50	37.7	105.6	73.7	15.6
**Percent of pelvic – anal distance**							
Vent distance	9.4	9.0	50	7.3	19.7	13.7	2.2
**Percent of pelvic-fin length**							
Length of axillary scale	21.5	33.1	50	18.8	35.5	27.2	3.8
**Percent of dorsal-fin depth**							
Depth of last unbranched dorsal-fin ray			49	83.3	93.8	89.0	2.3
Depth of first branched dorsal-fin ray			50	80.7	90.7	86.4	2.4
Depth of second branched dorsal-fin ray	84.5	80.6	50	69.4	97.6	76.9	4.1
Depth of third branched dorsal-fin ray	66.2	65.1	50	52.8	70.8	62.3	3.5
Depth of fourth branched dorsal-fin ray	54.5	52.5	50	40.8	57.6	49.4	3.1
Depth of fifth branched dorsal-fin ray	49.9	45.4	50	34.3	46.9	40.9	3.0
**Percent of disk length**							
Length of torus	22.5	22.0	50	11.2	24.3	16.6	2.7
Length of pulvinus	57.1	53.3	50	37.0	65.9	52.8	7.3
Length of labrum	20.4	24.8	50	13.1	47.9	30.5	8.9
Disc width	102.1	104.6	50	95.2	156.8	124.7	15.3
Width of mouth	121.3	118.0	50	111.7	183.4	151.1	16.1
Width of torus	71.5	70.6	50	68.6	97.6	84.1	6.9
Width of pulvinus	60.6	62.5	50	59.6	88.6	73.2	6.0

**Table 4.** Meristic character states in type specimens of *Garra makiensis* and *G. rothschildi* as well as additional specimens of *G. makiensis* from the Gotta River (AMNH 227323, MRAC 91-051-P-0044) and the Awash River drainage (deposited at NMW). Numbers in squared brackets referring to mean±SD. Information per specimen as in Table 1.

Character states	*G. makiensis*BMNH 1905.7.25.87 lectotype	*G. makiensis*BMNH 1905.7.25.88 paralectotype	*G. rothschildi*MNHN 1905-0246 syntype	*G. rothschildi*MNHN 1905-0247 syntype	*G. makiensis* Gotta River	*G. makiensis* Awash River
Unbranched dorsal-fin rays	4	4	4	4	3(5), 4(19) [3.8±0.4]	3(9), 4(30) [3.8±0.4]
Branched dorsal-fin rays	8	8	8	8	8(24) [8.0±0.0]	8(96) [8.0±0.0]
Unbranched anal-fin rays	3	3	3	3	3(24) [3.0±0.0]	3(46) [3.0±0.0]
Branched anal-fin rays	6	6	6	6	6(24) [6.0±0.0]	6(96) [6.0±0.0]
Unbranched pelvic-fin rays	1	1	1	1	1(24) [1.0±0.0]	1(81) [1.0±0.0]
Branched pelvic-fin rays	8	8	8	9	7(1) 8(12) [7.9±0.3]	8(81) [8.0±0.0]
Unbranched pectoral-fin rays	1	1	1	1	1(8) [1.0±0.0]	1(81) [1.0±0.0]
Branched pectoral-fin rays	15	16	15	15	14(1), 15(2), 16(2) [15.2±0.8]	13(1), 14(5), 15(20), 16(47), 17(8) [15.7±0.8]
Principal caudal-fin rays	17	17	17	17	17(24) [17.0±0.0]	17(46) [17.0±0.0]
Upper caudal-fin procurrent rays	8	9	8	8	7(2), 8(20), 9(1), 11(1) [8.1±0.7]	7(7), 8(27), 9(2) [7.9±0.5]
Lower caudal-fin procurrent rays	7	7	7	7	7(20), 8(4) [7.2±0.4]	6(14), 7(20), 8(2) [6.7±0.6]
Total number of lateral-series scales	37	38	39	38	38(4), 39(1) [38.2±0.5]	37(7), 38(31), 39(33), 40(10) [38.6±0.8]
Lateral-series scales to posterior margin of hypurals	34	36	36	36	36(4), 37(1) [36.2±0.5]	35(10), 36(32), 37(29), 38(9), 39(1) [36.5±0.9]
Total number of later-line scales	37	38	39	38	36(1), 38(4) [37.6±0.9]	36(3), 37(19), 38(27), 39(24), 40(8) [38.2±1.0]
Scale rows between lateral line – dorsal-fin origin	5	5	5	4	5(5) [5.0±0.0]	4(1), 5(80) [5.0±0.1]
Scale rows between lateral line – pelvic-fin origin	4	4	4	4	4(5) [4.0±0.0]	4(80), 5(1) [4.0±0.1]
Scale rows between lateral-line – anal-fin origin	4	4	4	4	4(3), 5(2) [4.4±0.6]	4(8), 5(42) [4.2±0.4]
Scale rows between lateral line – anus	5	5	5	5	5(1)	4(1), 5(48), 6(1) [5.0±0.2]
Circumpeduncular scales	16	16			16(5) [16.0±0.0]	16(81) [16.0±0.0]
Post-pelvic scales	9	9			6(3), 7(1), 9(1) [6.8±1.3]	6(3), 7(23), 8(22), 9(2) [7.5±0.7]
Anal scales	2	2	2	2	2(5) [2.0±0.0]	0(1), 1(16), 2(33) [1.6±0.5]
Total number of vertebrae	36	36	37	37	35(2), 36(19), 37(3) [36.0±0.5]	35(1), 36(4), 37(22), 38(18), 39(1) [37.3±0.8]
Abdominal vertebrae	20	20	21	20	20(8), 21(14), 22(2) [20.8±0.6]	20(4), 21(31), 22(11) [21.2±0.6]
Caudal vertebrae	16	16	16	17	14(2), 15(13), 16(9) [15.3±0.6]	14(1), 15(6), 16(24), 17(15) [16.2±0.7]
Predorsal abdominal vertebrae	10	10	10	10	9(1), 10(20), 11(1), 12(2) [10.2±0.6]	9(2), 10(23), 11(21) [10.4±0.6]
Preanal caudal vertebrae	3	3	2	3	1(9), 2(10), 3(5) [1.8±0.8]	1(4), 2(29), 3(13) [2.2±0.6]
Postanal vertebrae	13	13	14	14	12(1), 13(11), 14(12) [13.5±0.6]	12(1), 13(9), 14(28), 15(7), 16(1) [13.8±0.6]
Vertebrae between first pterygiophores of dorsal and anal fins	13	13	13	13	11(2), 12(11), 13(10), 14(1) [12.4±0.7]	12(13), 13(23), 14(10) [12.9±0.7]
Intermediate vertebrae	4	4	5	5	4(7), 5(12), 6(5) [4.9±0.7]	4(13), 5(28), 6(4) [4.8±0.6]
Supraneural bones	4	4	4	4	4(4) [4.0±0.0]	3(1), 4(10), 5(10) [4.4±0.6]

### Morphological analyses

Both PCA and DFA cluster type specimens of *G. makiensis* together with the Awash population identified as *G. makiensis*, while they are distinct from *G. aethiopica* and *G. dembeensis* (Fig. 3). Based on PCA the most distinguishing variables between the three species are dorsal-fin depth (% SL), number of unbranched and branched pectoral-fin rays, anal-fin depth (% SL), vent distance (% pelvic – anal distance), and pelvic-fin length (% SL) (Suppl. material 1: Table S4).

A similar pattern of morphological differences is supported by DFA. Variables that contribute most for discrimination of the samples are vent distance (% pelvic – anal distance), dorsal-fin depth (% SL), total number of lateral-series scales, and number of branched pectoral-fin rays. Predicted classifications for the samples from the Awash (*G. aethiopica*, *G. dembeensis*, *G. makiensis*) and the type specimens of *G. makiensis* were 100 % correct, with the exception of one specimen identified as *G. aethiopica* falling within the group of *G. dembeensis* (Suppl. material 1: Table S5).

### Taxonomy

#### Garra makiensis

Taxon classificationAnimaliaCypriniformesCyprinidae

(Boulenger, 1903)

82C0019E-5A32-51C3-B9F1-A0B682B0AF7B

**Figure 4.**
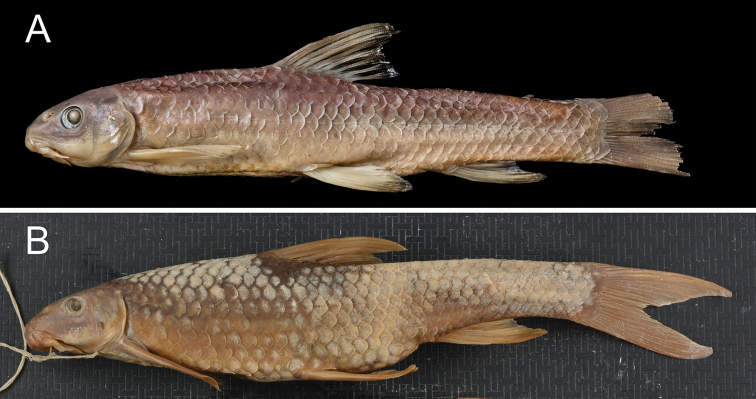
General appearance of *Garra makiensis*. **A**BMNH 1905.7.25.87, lectotype of *G. makiensis*, female, 67.1 mm SL, Maki [Meki] River, Ethiopia, The Trustees of the Natural History Museum, London, **B**MNHN-1905-0246, syntype of *G. rothschildi*, 135.3 mm SL, Gotta [Gota] River, Ethiopia, The Muséum national d’Histoire naturelle, Paris.

**Figure 5.**
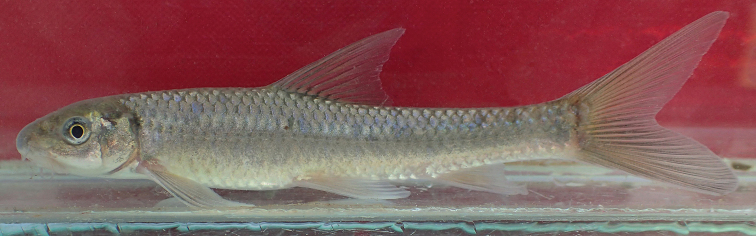
*Garra makiensis*, alive, NMW 99224, 52.0 mm SL, Awash River at Lafessa (S6). Photograph by W. Graf.

**Figure 6.**
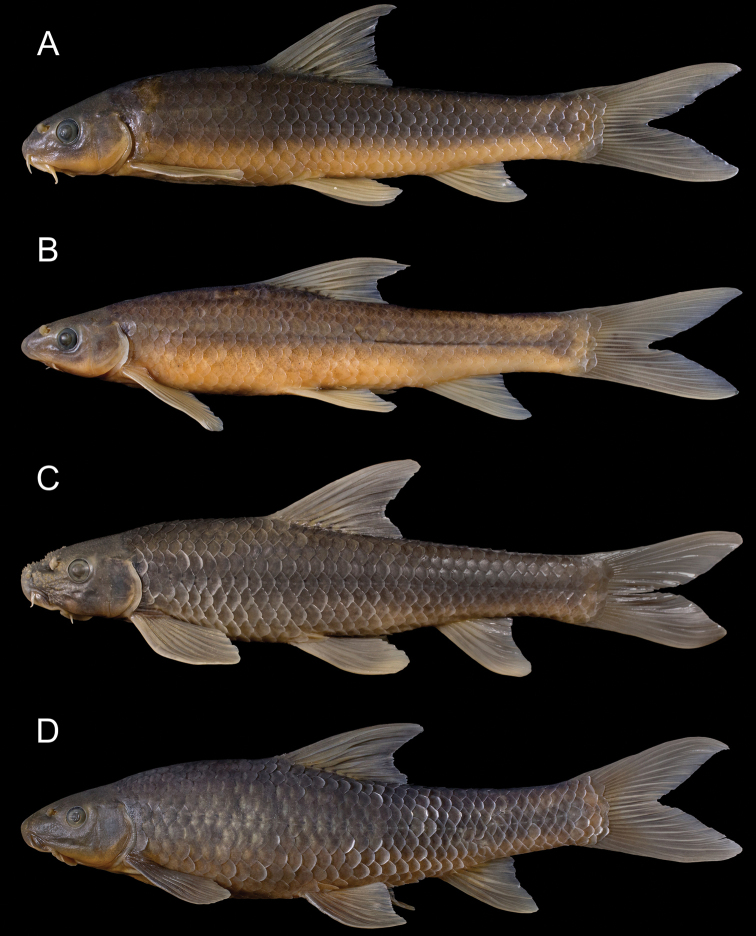
General appearance and morphological variability of *Garra makiensis* from the Awash River. **A**NMW 99485, female, 85.8 mm SL, Adayitu (S13), **B**NMW 99485, female, 82.2 mm SL, Adayitu (S13), **C**NMW 99491, male, 106.1 mm SL, Dubti (S14), **D**NMW 99504, female, 147.9 mm SL, Jara River (T3).

**Figure 7.**
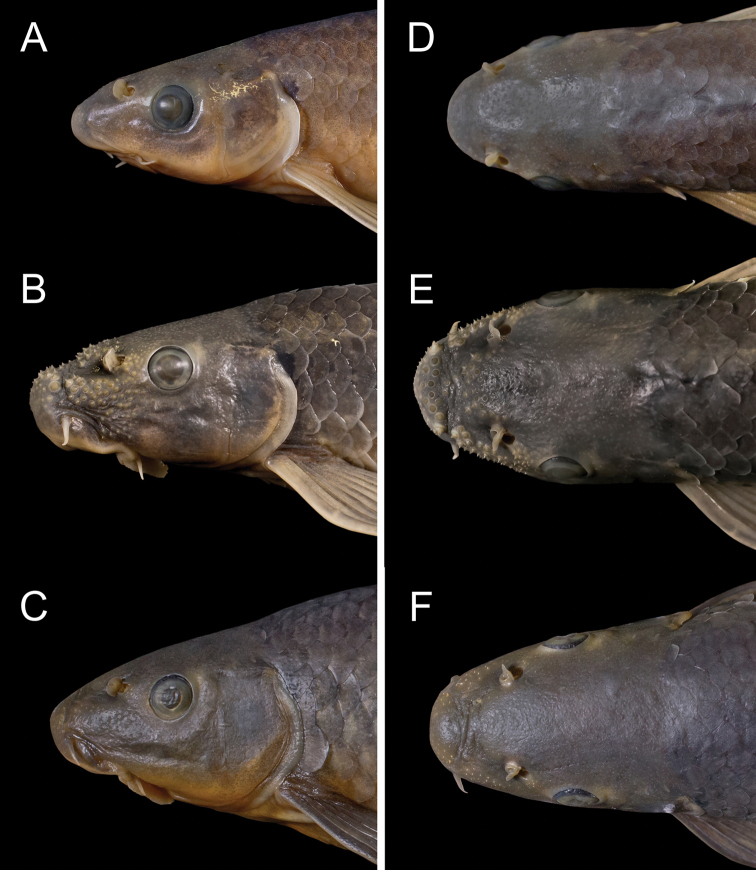
Lateral and dorsal view of head, in *Garra makiensis* from the Awash River. **A** and **D** same specimen as in Fig. 6B, **B** and **E** same specimen as in Fig. 6C, **C** and **F** same specimen as in Fig. 6D.

**Figure 8.**
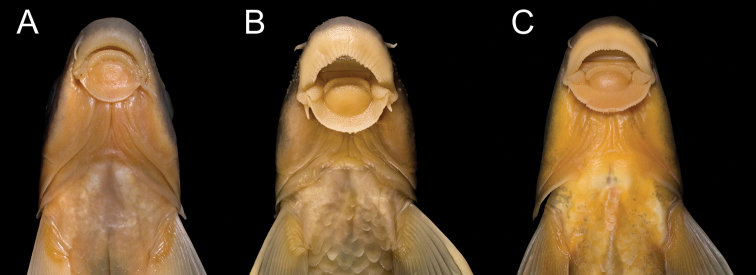
Ventral view of head, in *Garra makiensis* from the Awash River. **A** same specimen as in Fig. 6B, **B** same specimen as in Fig. 6C, **C** same specimen as in Fig. 6D.

**Figure 9.**
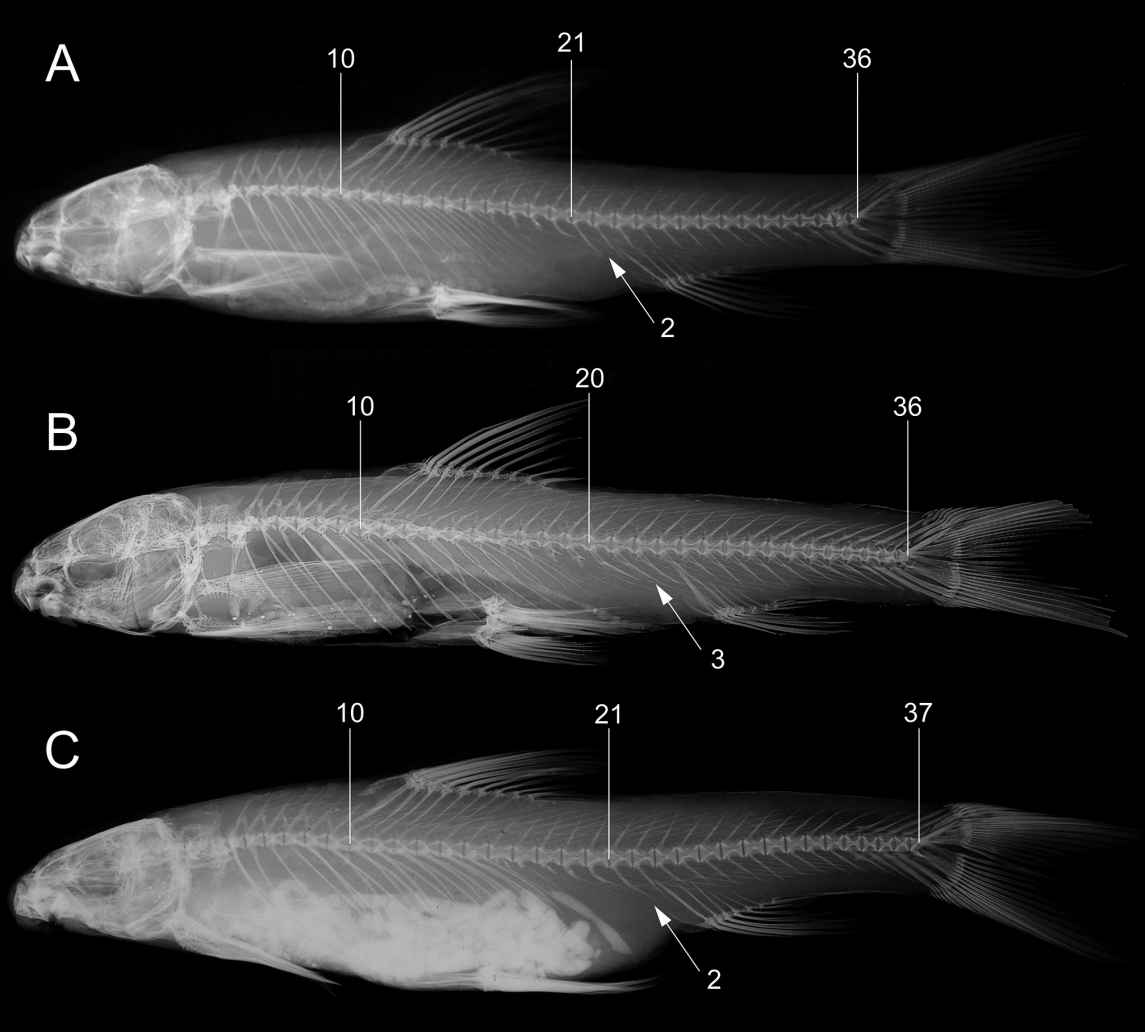
Axial skeletons and supraneural bones in *Garra makiensis*. **A**NMW 99223, 56.0 mm SL, Awash River at Wonji (S7), 10 showing last predorsal abdominal vertebra and 21 last abdominal vertebra, total vertebrae 36:21+15, **B** lectotype of *G. makiensis*, same specimen as in Fig. 4A, 10 showing last predorsal abdominal vertebra and 20 last abdominal vertebra, total vertebrae 36:20+16, The Trustees of the Natural History Museum, London, **C** syntype of *G. rothschildi*, same specimens as in Fig. 4B, 10 showing last predorsal abdominal vertebra and 21 last abdominal vertebra, total vertebrae 37:21+16, The Muséum national d’Histoire naturelle, Paris. Arrows showing position and numbers of preanal caudal vertebrae.

Figures 4 , 5 , 6 , 7 , 8 , 9

Discognathus makiensis Boulenger, 1903:330 (type locality: Maki [Meki] River, Ethiopia), Fig. 4A
Discognathus rothschildi Pellegrin, 1905:291 (type locality: Gotta [Gota] River, Ethiopia), Fig. 4B

##### Material examined.

Comparative material from the Awash River drainage (including the Gotta River sub-drainage) is listed in Table 1.

##### Identification.

See Figs 5–8 for general appearance of *G. makiensis* from the Awash River; Fig. 7 for tubercles on head; Fig. 8 for scales on chest and shape of gular disc; and Fig. 9 for axial skeleton and shape of supraneural bones. Tubercles on scales and the pectoral fin, and scale pattern on ventral side are shown in Suppl. material 2: Figs S1, S2. Measurements and counts are given in Tables 3, 4.

Longest examined specimen 147.9 mm SL (female, NMW 99504). Body elongated, moderately compressed, more in the caudal region. Shape of body and head very variable. Dorsal head profile slightly convex, its transition to back usually smooth, in few specimens with a slight nuchal hump. In most specimens, predorsal back outline rises gently, slightly convex or straight, to dorsal-fin origin. Postdorsal profile slightly concave to caudal-fin origin. Caudal peduncle almost twice as long as its minimal depth. The vent is close to the anal-fin origin (7.3–19.7 % of pelvic – anal distance). Head usually as long as body depth at dorsal-fin origin. Head depth at nape shorter than head length. Snout blunt and longer than orbit – operculum distance. Transverse groove weakly developed (absent in some specimens); transverse lobe separated from lateral field by a shallow groove (or without groove). Two deep grooves originating above anterior barbel, posteriorly not connected; at posterior end of upper groove a patch of few tubercles in some specimens (Fig. 7).

Tubercles on snout and head in both males and females (smallest specimen with tubercles: 45.4 mm SL, Awash River, S14), but often completely absent or rudimentary developed (Figs 6, 7). Transverse lobe with large conical tubercles; tubercles extending to lateral surface and the area between anterior rim of eyes and nostrils. Depressed rostral margin usually without tubercles; in some specimens few and irregularly placed. Anterior extremity of the ethmoid field often elevated from depressed rostral surface and covered with large tubercles, especially in anterior region. Small tubercles are commonly spread on the frontal and occipital regions, sometimes extending to the operculum (Fig. 7 and Suppl. material 2: Fig. S1). In few specimens (n = 7), small circular tubercles on scales in the predorsal region and the lateral side of the abdominal region (above lateral line). A single specimen (NMW 99231, male, 91.2 mm SL, Awash River, S10) with tubercles on dorsal side of the pectoral fins (at fin membranes) (Suppl. material 2: Fig. S1).

Gular disc well-developed but often variable in size and shape (Fig. 8). Its width greater than its length. Width of torus less than disc length. Pulvinus wider than long and with few papillae. Labrum well-developed and longer than torus. Width of mouth usually less than snout length. Abundant papillae on rostral cap, torus, labellum and labrum. Rostral cap with invecked ventral margin. Two pairs of barbels, their length usually shorter than eye diameter; anterior barbel slightly longer or about equal to posterior barbel.

Dorsal fin with 3 or 4, commonly 4, unbranched and 8 branched rays, its last unbranched ray is the longest (89.0 % of dorsal-fin depth); length of first branched ray 86.4 % of dorsal-fin depth; second branched ray much shorter (76.9 % of dorsal-fin depth). Pelvic fin with a single unbranched ray and 7–9, commonly 8, branched rays; pelvic splint present. Long axillary scale at base of pelvic fin, its length 18.8–35.5 % of pelvic-fin length. Pectoral fin with a single unbranched ray and 13–17, commonly 16, branched rays. Caudal fin forked with 2+17 principal rays. Upper procurrent rays 7 (9), 8 (49) or 9 (3), lower procurrent rays 6 (14), 7 (42) or 8 (6).

Lateral line complete and going along midline. Total lateral-series with 37–40, commonly 38, scales. Lateral-series scales to posterior margin of hypurals 35–39, commonly 36. Transversal scale rows between lateral line and dorsal-fin origin 4 or 5, commonly 5; and 4–6, commonly 5 between lateral line and anal-fin origin. Chest, belly, postpelvic and predorsal regions fully scaled. Scales on chest usually deeply embedded (Fig. 8 and Suppl. material 2: Fig. S2); predorsal scales irregularly arranged. Between anal-fin origin and anus 0 (1) 1 (16) or 2 (42) scales; and 6 (6), 7 (24), 8 (22) or 9 (3) scales in postpelvic region. Circumpeduncular scale rows 16.

Total vertebrae 35–39, commonly 37; with abdominal vertebrae 20–22; predorsal abdominal vertebrae 9–12; caudal vertebrae 14–17; and 11–14 vertebrae between first pterygiophores of dorsal and anal fins. Most frequent vertebral formulae 21+16 (19, n = 74). Supraneural bones 3–5 (commonly 4 (16) or 5 (10), n = 27), first two square shaped and last two to three in front of dorsal fin elongated and largest (Fig. 8).

##### Morphological variability.

Similar to other *Garra* species in Africa and the Arabian Peninsula (Krupp 1983; Golubtsov et al. 2012; Englmaier 2018), we found considerable morphological variability in *G. makiensis* (Fig. 6). Based on our data, we cannot confirm the presence of a sexual dimorphism, but the largest specimens were females (> 140 mm SL), and more males with prominent tuberculation on snout and head were found. Though few specimens were examined, and samples were collected during dry season only, our data suggest that body shape and tuberculation in *G. makiensis* might be (directly or indirectly) related to abiotic habitat characteristics. Specimens with a more slender body shape and without (or reduced) tubercles on snout and head (Fig. 6B) were caught in low flow velocity habitats, whereas deep bodied specimens with large conical tubercles on snout and head (Fig. 6C) exclusively occurred in high flow velocity habitats over coarse substrate. Intermediate morphs (Fig. 6A) and large growing specimens with reduced tubercles (Fig. 6D) were occasionally found.

##### Colouration.

In life (Fig. 5): Body colour usually light grey, above lateral line often pale-brown or blueish iridescent and darker than below. Head yellowish brown, mouth and ventral side cream. Iris white and yellow. Some individuals show an indistinct, roundish, dark blotch at posteriormost caudal peduncle. At anteriormost lateral line (behind upper edge of operculum) a small dark (rarely blueish iridescent, never red) blotch, not extending on gill cover. Fin membranes usually hyaline, sometimes light grey or yellowish; on caudal fin often light orange. Fin rays hyaline or pale. Dorsal fin with four to six indistinct black blotches at base of branched rays (strongest between 3^rd^ and 6^th^ branched rays).

In formalin (initial fixation) and later transferred to 75 % ethanol (Figs 6–8): Specimens usually light to dark grey, sometimes cream or brownish; darker above lateral line; ventral side cream to yellowish or orange. Back usually dark greyish; head brownish grey. Dark mid-lateral stripe usually of increasing intensity at caudal peduncle, often forming an indistinct blotch at posteriormost caudal peduncle. Fins pale, anterior part of caudal-fin base brownish. Indistinct black blotches at base of branched dorsal-fin rays (strongest between 3^rd^ and 6^th^ branched rays).

##### Habitat.

**Figure 10.**
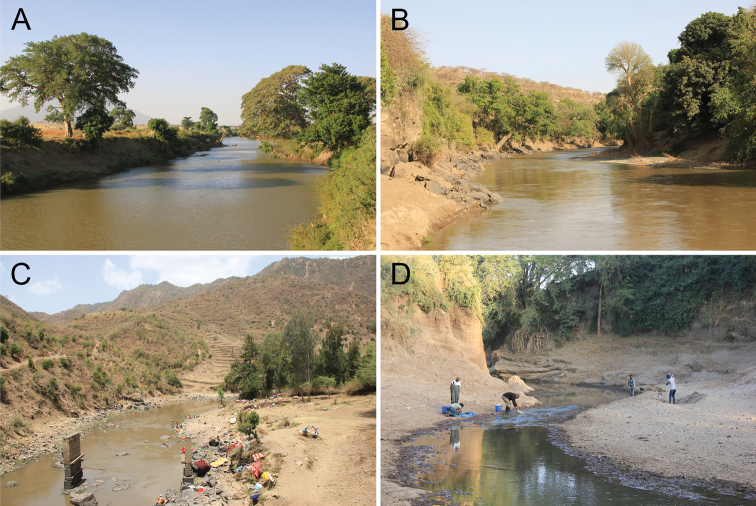
Habitat of *Garra makiensis* in the Awash River drainage and sampling site in the Lower Meki River where *G. makiensis* was absent. **A** Awash River at Lafessa (S6, 1,608 m a.s.l.), **B** Awash River at Yimre (S10, 797 m a.s.l.), **C** Middle Borkana River (T3, 1,417 m a.s.l.), **D** Lower Meki River, upstream of Meki town (1,663 m a.s.l.).

*Garra makiensis* was sampled from the mainstem Awash River and its tributaries (Mille River (T1), Borkana River (T3) and Jara River (T4)) (Figs 1, 10 and Suppl. material 1: Table S1). The altitude ranged from 1,608 m a.s.l. (8°23'16"N, 38°54'30"E, S6) to 338 m a.s.l. (11°30'50"N, 41°38'51"E, S16). Specimens were collected from shoreline habitats, deeper stretches of the main channel, side channels, stagnant water bodies of the floodplains and lacustrine habitats (e.g., lakes Yardi and Gamari, Koka Reservoir); both low-flow and high-flow velocity habitats were inhabited. Substrate composition ranged from silt and sand to coarse stony substrate. The water was usually turbid (suspended solids); water temperature ranged from 21.1 °C to 31.9 °C; conductivity was between 286.7–1,710.3 μS cm^-1^; and dissolved oxygen was close to saturation (65.1–124.1 %) (Englmaier et al. 2020b).

##### Distribution.

*Garra makiensis* is endemic to Ethiopia where it is found in endorheic drainages (Awash (including the Gotta River sub-drainage) and Meki) of the Northern and Central MER (Fig. 1) (Golubtsov et al. 2002). It is absent from the headwaters and was found characteristic for the middle and lower sections of the Awash River (Englmaier et al. 2020b). In the current study, we cannot confirm the presence of *G. makiensis* in the Meki River, its type locality. The Meki drainage is highly altered by human impacts (e.g., water abstraction, sand mining) and the last records of *G. makiensis* in this drainage date back to 1984 (Golubtsov et al. 2002). The extended distribution range reported by Stiassny and Getahun (2007), including the southern part of the MER, the Blue and White Nile drainages and the Omo River drainage, contains uncertainty and needs clarification (Wakjira and Getahun 2017).

### Molecular analyses

**Figure 11.**
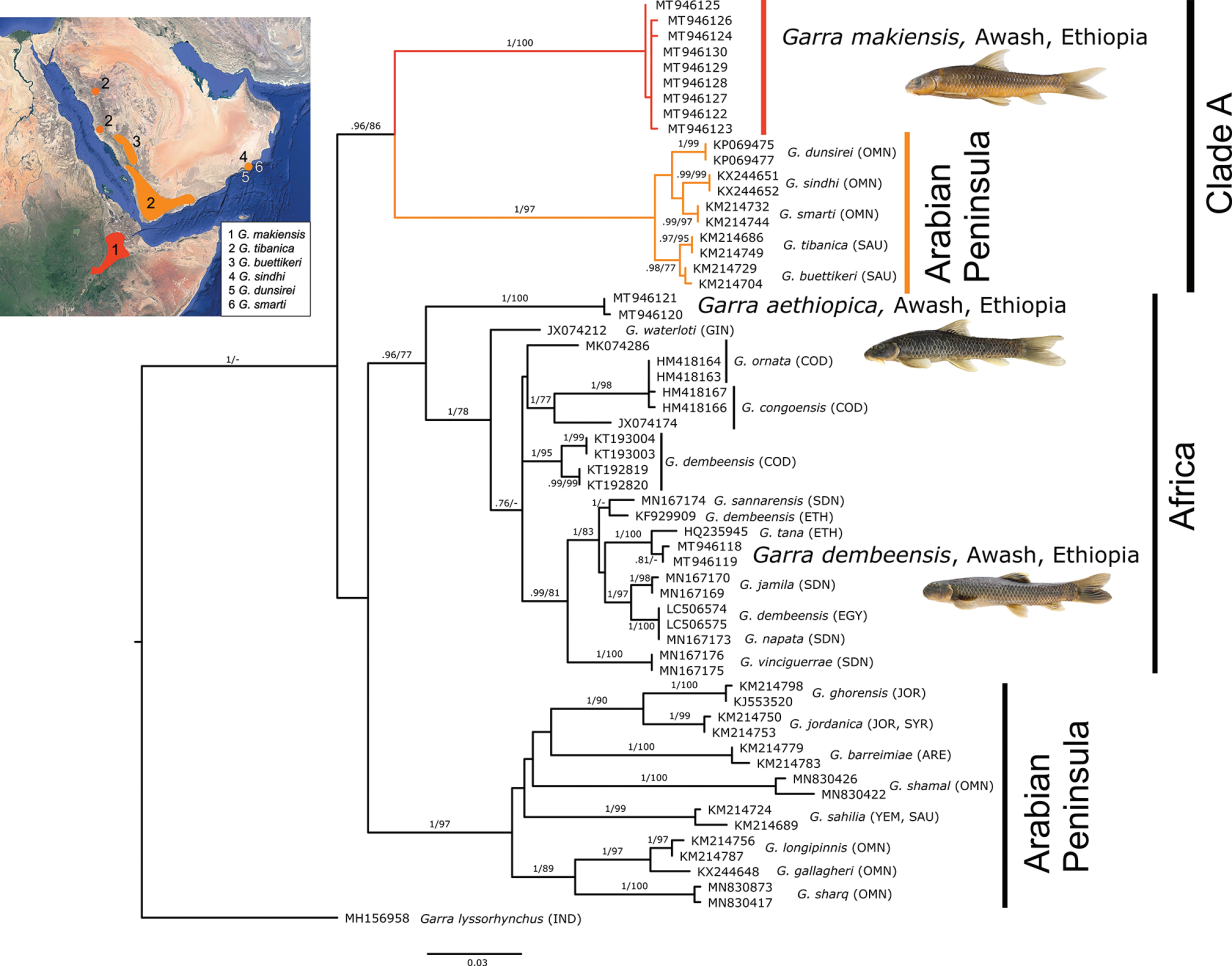
ML tree based on CO1 sequences (611 bp; MT946130 paralectotype of *G. makiensis*, 451 bp), showing phylogenetic relationships of *Garra* species from Awash with congeneric lineages from Africa and the Middle East. Numbers above branches represent Bayesian posterior probability (BPP) of BI/bootstrap values (bs) of ML. Only values above .70/70 are shown. On the top left, small map showing the distribution of Clade A in Ethiopia and the Arabian Peninsula; distribution of species on the Arabian Peninsula according to Krupp (1983) (source of map: Google Earth Pro v. 7.3). Colours of branches in Clade A correspond to those in the map. Countries of origin of the sequences are represented with ISO codes. The topology of the BI analysis is given in Suppl. material 2: Fig. S4.

The alignment used for BI and ML phylogenetic reconstructions comprised 59 CO1 sequences of a length of 611 bp, and one sequence (BMNH 1905.7.25.88, *G. makiensis*, paralectotype) with a length of 451 bp. The alignment included nine individuals of *G. makiensis*, two individuals of *G. dembeensis* and two individuals of *G. aethiopica*, all from the Awash River. Forty-six other sequences of *Garra* species from Africa and the Middle East were included to resolve the phylogenetic relationships of the Awash species (Fig. 11).

*Garra makiensis* clusters together with species from the south-western Arabian Peninsula (*G. tibanica* Trewavas, 1941, *G. buettikeri* Krupp, 1983, *G. dunsirei* Banister, 1987, *G. smarti* and *G. sindhi*) forming a monophyletic group (Bayesian posterior probability, BPP 0.96; bootstrap value, bs 86; Clade A, Fig. 11). The lineage of *G. makiensis* appears as a strongly supported sister lineage to all remaining species within Clade A (BPP 1; bs 100). Pairwise distances between *G. makiensis* and Arabian Peninsula species range from 9.51 % to 10.23 % (Suppl. material 1: Table S7). *Garra makiensis* is clearly distinct from congeners in the Awash River. Pairwise distance between *G. makiensis* and *G. aethiopica* is 9.90 % and 9.78 % between *G. makiensis* and *G. dembeensis*. The paralectotype of *G. makiensis* clusters together with our samples of *G. makiensis* from the Awash River (p-distance 0.08 %, Suppl. material 1: Table S7), corroborating the identification of the Awash population as *G. makiensis*.

*Garra aethiopica* forms a distinct, monophyletic lineage within a cluster of other African *Garra* (BPP 1; bs 100). Pairwise distance between *G. aethiopica* and all other African congeners range from 6.46 % to 8.27 %. Individuals of *G. dembeensis* from the Awash River appear as a sister clade to *G. tana* Getahun & Stiassny, 2007 (BPP 1; bs 100) (Lake Tana, Upper Blue Nile drainage) and do not cluster together with other sequences named as *G. dembeensis* from the Congo (KT193003, KT193004, KT192819, KT192820) and the Nile River (KF929909, LC506574, LC506575) drainages. Pairwise distance between *G. dembeensis* from Awash River and *G. tana* is 1.06 % (Suppl. material 1: Table S7).

In summary, mitochondrial CO1 data provide support that *G. makiensis* is more closely related to *Garra* species in the south-west of the Arabian Peninsula than to congeners from Africa, as all of them belong to a different, well-supported, monophyletic clade (BPP 0.96; bs 77). Below we provide a morphological comparison of *G. makiensis* with closely related species of Clade A (Fig. 11).

### Comparison of *G. makiensis* with congeners of Clade A.

*Garra* species of Clade A (Fig. 11) are currently known only from the north-east of Ethiopia (*G. makiensis*) and the south-west of the Arabian Peninsula (Trewavas 1941; Balletto and Spanò 1977; Krupp 1983; Banister 1987; Golubtsov et al. 2002; Krupp and Budd 2009; Lyon et al. 2016). For morphological comparison we combined all specimens of *G. makiensis* from Ethiopia, including the type specimens of *G. makiensis* and *G. rothschildi*, into one sample. No original material of *G. buettikeri*, *G. dunsirei*, *G. smarti*, and *G. sindhi* was examined and we refer to published data (original descriptions: Trewavas (1941), Krupp (1983), Banister (1987), Krupp and Budd (2009), and Lyon et al. (2016)) for comparison. *Garra tibanica* includes several subspecies (Balletto and Spanò 1977) of unknown systematic relationship and taxonomic status. We, therefore, refer to *G. tibanica* as described by Trewavas (1941) and present new data on axial skeleton elements for the type specimens (Suppl. material 1: Table S8).

*Garra makiensis* can be distinguished from *G. buettikeri* (eastern side of the Asir mountains, draining to the Wadi ad-Dawasir, Saudi Arabia) by 4–5 scales between the lateral line and the dorsal-fin origin (vs. 6.5–8.5); 16 circumpeduncular scales (vs. 18–20); and caudal peduncle length 17–23 % SL (vs. 15–19 % SL). The number of lateral-series scales largely overlap (37–40, mode 38 vs. 36–39, mode 37), but the lowest count, 36 (n = 10), recorded in *G. buettikeri* was not found in *G. makiensis*. Analysis of mitochondrial CO1 place *G. buettikeri* closest to *G. tibanica* (p-distance 0.57 %; Suppl. material 1: Table S7) (Hamidan et al. 2014).

*Garra makiensis* differs from *G. tibanica* (coastal Wadi Tiban drainage, Yemen) by 37–40, commonly 38, scales in the lateral series (vs. 32–34); 35–39, commonly 37, total vertebrae (vs. 32–33); 20–22 abdominal vertebrae (vs. 19); 14–17 caudal vertebrae (vs. 13–14); and 11–14 vertebrae between first pterygiophores of dorsal and anal fins (vs. 10–12). *Garra makiensis* shares with *G. tibanica* such characters as a completely scaled chest and belly (Trewavas 1941: 12); the pattern of nuptial tubercles on snout (Krupp 1983: fig. 38); and a short distance between anus and anal-fin origin (7.3–19.7 vs. 16.7–20.0 % of pelvic – anal distance) (Trewavas 1941).

*Garra makiensis* clearly differs from *G. dunsirei* (sinkhole at Tawi Atair, Dhofar Region, Oman) by the presence of scales on chest and belly (vs. reduced scales on ventral side), 16 circumpeduncular scales (vs. 12); 35–39, commonly 37, total vertebrae (vs. 36 or 37); width of gular disc wider than its length (vs. width of gular disc slightly smaller than its length); and eye diameter 4–6 % SL (vs. 3–4 % SL). Mitochondrial CO1 data place *G. dunsirei* close to *G. smarti* and *G. sindhi* (p-distances 1.47 % and 1.96 % respectively; Suppl. material 1: Table S7) from the same geographic area (Lyon et al. 2016).

*Garra makiensis* is distinct from *G. smarti* (Wadi Hasik, Dofar Region, Oman) by 37–40, commonly 38, scales in the lateral series (vs. 34–35, commonly 34); 35–39, commonly 37 total vertebrae (vs. 32–34, mode 33); 20–22 abdominal vertebrae (vs. 19–20); 14–17 caudal vertebrae (vs. 13–15); width of gular disc wider than its length (vs. width of gular disc usually longer than its width); and anal fin depth 19–22 % SL (vs. 16–17 % SL).

*Garra makiensis* is further distinguished from *G. sindhi* (Lower Wadi Andhur, Dofar Region, Oman) by 13–17, commonly 16, branched pectoral fin rays (vs. 12); 37–40, commonly 38, scales in the lateral series (vs. 36); 34–39 scales in the lateral-line series to posterior margin of hypurals (vs. 34); and anal-fin depth 19–22 % SL (vs. 14–20 % SL). Both species are similar by their prominent axillary scale; deeply embedded scales on chest; and commonly 2 scales between anus and anal-fin origin.

## Biogeographical aspects

The distribution ranges and systematic relationships of African *Garra* species are still poorly investigated. Our morphological and mtDNA data suggest 1) a palaeohydrological connection between the Awash River drainage and the lakes of the Central MER as *G. makiensis* is known from both drainage systems (see also Englmaier et al. 2020a and references therein); 2) the distinctiveness of *G. makiensis* in comparison to *G. aethiopica* and *G. dembeensis* from the Awash, suggesting a different evolutionary or colonisation history of *Garra* species in the region; and 3) a closer relationship between *G. makiensis* and *Garra* species in the south-west of the Arabian Peninsula than to African congeners.

Biogeographical similarities between the Horn of Africa and the Arabian Peninsula are evident for different animal groups (e.g., Pook et al. 2009; Zinner et al. 2009; Portik and Papenfuss 2012; 2015; Šmíd et al. 2013; Gilbert et al. 2014; Garcia-Alix et al. 2016; Yanai et al. 2020). Though dispersal routes across the Bab-al-Mandab Strait have been proposed by several studies (Šmíd et al. 2013; Stewart and Murray 2017), the exact timing is still controversial (Fernandes et al. 2006). Geological data provide evidence that the formation of the southern Red Sea rift section began in the early Oligocene with a first culmination from the upper Oligocene to the lower Miocene (30–23 Ma), followed by a geologically well-established reconnection period of Africa and Arabia at the Bab-al-Mandab-strait in the uppermost Miocene and Pliocene (10–5 Ma) (Bosworth et al. 2005; Autin et al. 2010). Postulated land bridge periods in younger times are not supported (Fernandes et al. 2006).

The restricted distribution of *G. makiensis* in the Northern and Central MER, and its close relationship to *Garra* species in the south-west of the Arabian Peninsula (based on CO1 sequence data) may support the hypotheses of dispersal events and vicariance around the southern Red Sea area. However, based on our mtDNA data, *G. makiensis* is currently the only known African species of Clade A (Fig. 11) and further investigations of the coastal drainages in the Horn of Africa are needed to clarify ichthyofaunal similarities across the Red Sea. Several examples can be found in literature: 1) a close affinity of *G. tibanica* with *G. blanfordii* (Boulenger, 1901) from coastal drainages in Eritrea was suggested by Trewavas (1941); 2) Menon (1958, 1964) placed *G. ethelwynnae* Menon, 1958 from Salamona (Eritrea) close to *G. tibanica*; and 3) Stiassny and Getahun (2007) synonymised *G. tibanica* and *G. brittoni* Trewavas, 1941 with *G. quadrimaculata* (Rüppell, 1835) from the Ethiopian Highlands. Furthermore, the Somalian cavefish *G. andruzzii* (Viciguerra, 1924) might reflect early dispersal events among *Garra* species in Africa (Yang et al. 2012).

The high genetic diversity and tree topology observed, not only within Clade A but in the whole studied dataset, suggest a complex evolutionary history and different evolutionary rates within the focal taxa. A more thorough sampling and deeper genome-level sequencing are needed to clarify the phylogenetic relationships and taxonomic status of several African *Garra* species.

In summary, we provide new data on morphology, mtDNA, and distribution of *G. makiensis* in Ethiopia. By introducing a wide set of morphological characters, we hope to support further morphological comparisons among *Garra* species in African and beyond. The CO1 sequence of the historic paralectotype of *G. makiensis* demonstrates that the use of historic museum material in phylogenetic analyses and species identification provides an invaluable potential for taxonomic studies, in particular in phenotypically variable groups. In the future, further research on African *Garra* is needed to clarify phylogenetic relationships, evolutionary history, and intraspecific morphological plasticity, including the variability of tubercles observed in the present study.

## Data availability

Data and alignments are available from the supplementary material and from the corresponding author upon reasonable request. Newly obtained sequences are deposited in GenBank under accession numbers MT946118–MT946130.

## Supplementary Material

XML Treatment for Garra makiensis

## Supplementary materials

Supplementary material 1 Supplementary tables Data type species data Explanation note **Table S1.** Sampling sites in the Northern and Central Main Ethiopian Rift. *Garra makiensis* was found only at sites S6–S16, T1 and T3–T4 in the Awash River drainage. **Table S2.** List of character states (morphometric characters (point to point measurements), and meristic characters) for the genus *Garra* used in the present study. **Table S3.** Primary data (morphometric and meristic) used in DFA (Fig. 3). Data for *Garra dembeensis* and *G. aethiopica* were taken from Englmaier (2018). Measurements and counts refer to those defined in Suppl. material 1: Table S2. **Table S4.**PCA statistics, **A** Communalities, **B** Total Variance Explained, **C** Component Matrix. **Table S5.**DFA statistics, **A** Tests of equality of group means, **B** Structure matrix, **C** Canonical Discriminant Function Coefficients, **D** Classification Function Coefficients, **E** Classification results. **Table S6.** Voucher specimens included in the present study. **Table S7.** Mean genetic distances based on CO1 sequences (uncorrected p-distances), in %. The comparison of the paralectotype to all other samples (in yellow) is based on a sequence length of 451 bp; comparisons among other samples do not include the paralectotype and are based on a sequence length of 611 bp. **Table S8.** Counts of axial skeleton elements in type specimens of *Garra tibanica*. https://binary.pensoft.net/file/471376This dataset is made available under the Open Database License (http://opendatacommons.org/licenses/odbl/1.0/). The Open Database License (ODbL) is a license agreement intended to allow users to freely share, modify, and use this Dataset while maintaining this same freedom for others, provided that the original source and author(s) are credited.Gernot K. Englmaier, Nuria Viñuela Rodríguez, Herwig Waidbacher, Anja Palandačić, Genanaw Tesfaye, Wolfgang Gessl, Paul Meulenbroek

Supplementary material 2 Supplementary figures Data type species data Explanation note **Figure S1.** Examples of tubercles and position of axillary scale in *Garra makiensis*, NMW 99231, 91.2 mm SL, male, Awash River at Yimre (S10). **A** tubercles on snout, head, and scales (above lateral line), **1** showing roundish tubercles extending from frontal region to operculum, **2** showing long axillary scale at base of pelvic fin; **B** conical tubercles on pectoral fin, **3** and **4** showing position of conical tubercles on fin membranes between fin rays. The presence of tubercles on the pectoral fin among African *Garra* species was only reported for *G. ornata* (Nichols & Griscom, 1917) so far (Getahun 2000). However, in this species tubercles were reported from the “underside of the pectoral fins” (Getahun 2000: 121). **Figure S2.** Ventral side of *Garra makiensis* from the Awash River with a completely scaled chest, belly, and postpelvic region (scales on chest usually deeply embedded). **A**NMW 99504, 147.9 mm SL, Jara River (T3) **B**NMW 99491, 106.1 mm SL, Dubti (S14); **1** showing scales on chest, **2** showing presence of a single unbranched pectoral-fin ray in *G. makiensis*. **Figure S3.** Ventral side of *Garra makiensis* from the Gotta River. **A**MNHN-1905-0247, syntype of *G. rothschildi*, 108.7 mm SL, The Muséum national d’Histoire naturelle, Paris; **B**AMNH 227323, Errer Gota [Gotta] River, Eastern side of Errer town, pools near main road, Hararge, Ethiopia (09°30'N, 41°15'E), The American Museum of Natural History. Arrows showing embedded scales on chest. **Figure S4.**BI tree based on CO1 sequences. https://binary.pensoft.net/file/471377This dataset is made available under the Open Database License (http://opendatacommons.org/licenses/odbl/1.0/). The Open Database License (ODbL) is a license agreement intended to allow users to freely share, modify, and use this Dataset while maintaining this same freedom for others, provided that the original source and author(s) are credited.Gernot K. Englmaier, Nuria Viñuela Rodríguez, Herwig Waidbacher, Anja Palandačić, Genanaw Tesfaye, Wolfgang Gessl, Paul Meulenbroek
