# Supplementary material for: New data on Garra makiensis (Cyprinidae, Labeoinae) from the Awash River (Ethiopia) with remarks on its relationships to congeners on the Arabian Peninsula
Source: Zookeys. 2020 Nov 4;984:133–63. doi: 10.3897/zookeys.984.55982 (PMC7658184; doi:10.3897/zookeys.984.55982)
Supplement: Supplementary material 2 — Supplementary figures [file zookeys-984-133-s002.docx]

**Supplementary material 2**


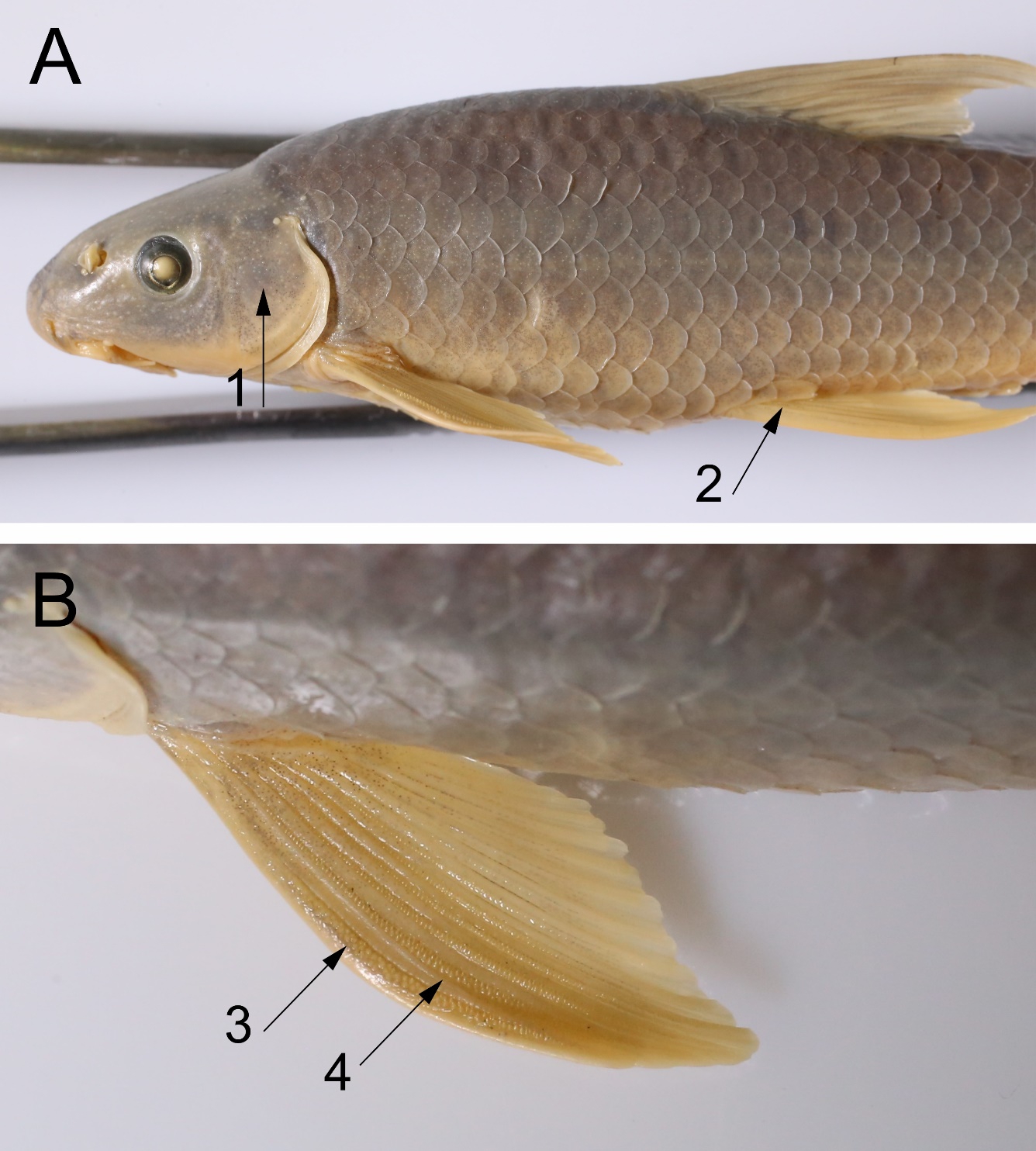


**Figure S1.** Examples of tubercles and position of axillary scale in *Garra makiensis*, NMW 99231, 91.2 mm SL, male, Awash River at Yimre (S10). **A** tubercles on snout, head, and scales (above lateral line), **1** showing roundish tubercles extending from frontal region to operculum, **2** showing long axillary scale at base of pelvic fin; **B** conical tubercles on pectoral fin, **3** and **4** showing position of conical tubercles on fin membranes between fin rays. The presence of tubercles on the pectoral fin among African *Garra* species was only reported for *G. ornata* (Nichols & Griscom, 1917) so far (Getahun 2000). However, in this species tubercles were reported from the “underside of the pectoral fins” (Getahun 2000: 121).


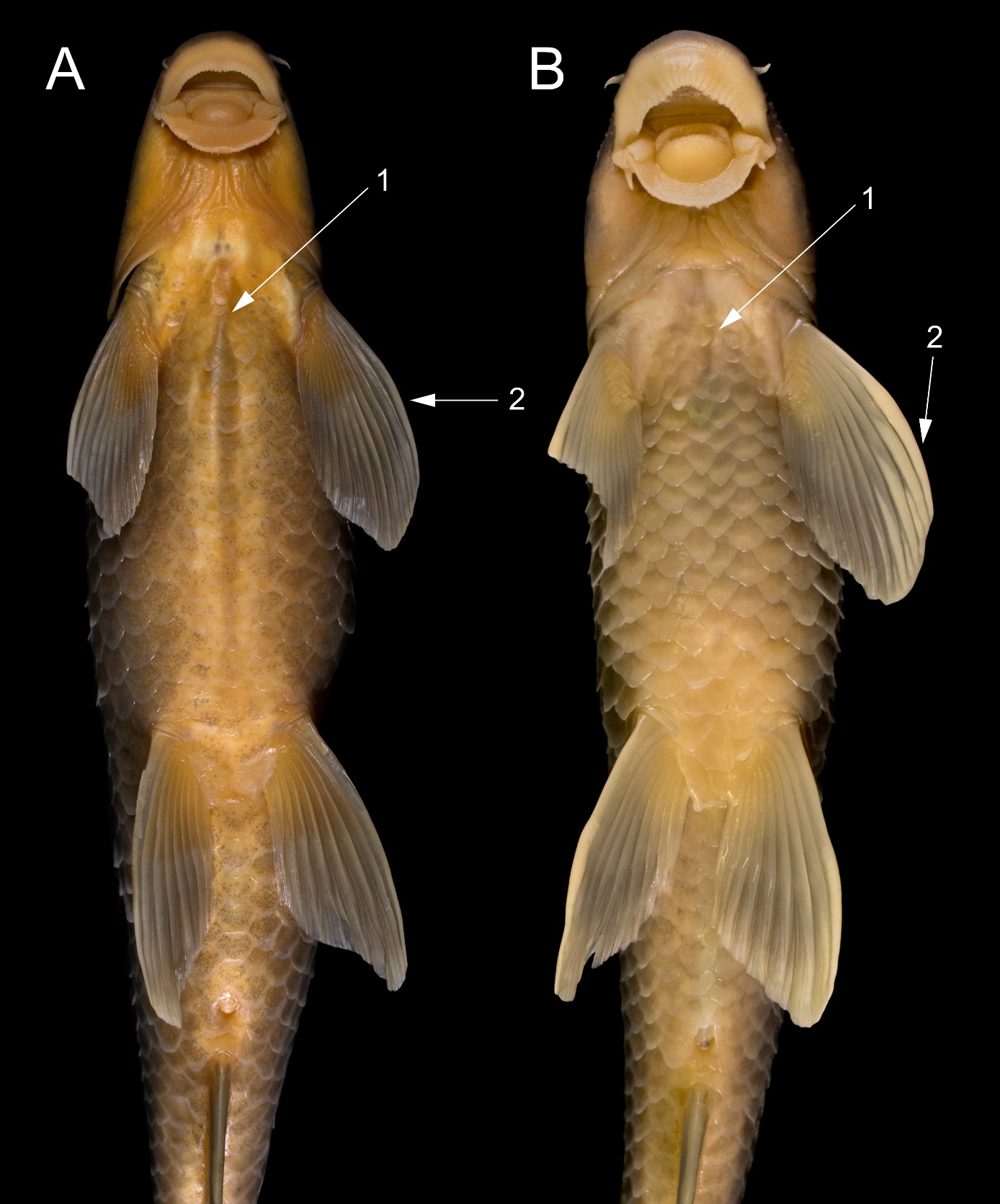


**Figure S2.** Ventral side of *Garra makiensis* from the Awash River with a completely scaled chest, belly, and postpelvic region (scales on chest usually deeply embedded). **A** NMW 99504, 147.9 mm SL, Jara River (T3) **B** NMW 99491, 106.1 mm SL, Dubti (S14); **1** showing scales on chest, **2** showing presence of a single unbranched pectoral-fin ray in *G. makiensis*.


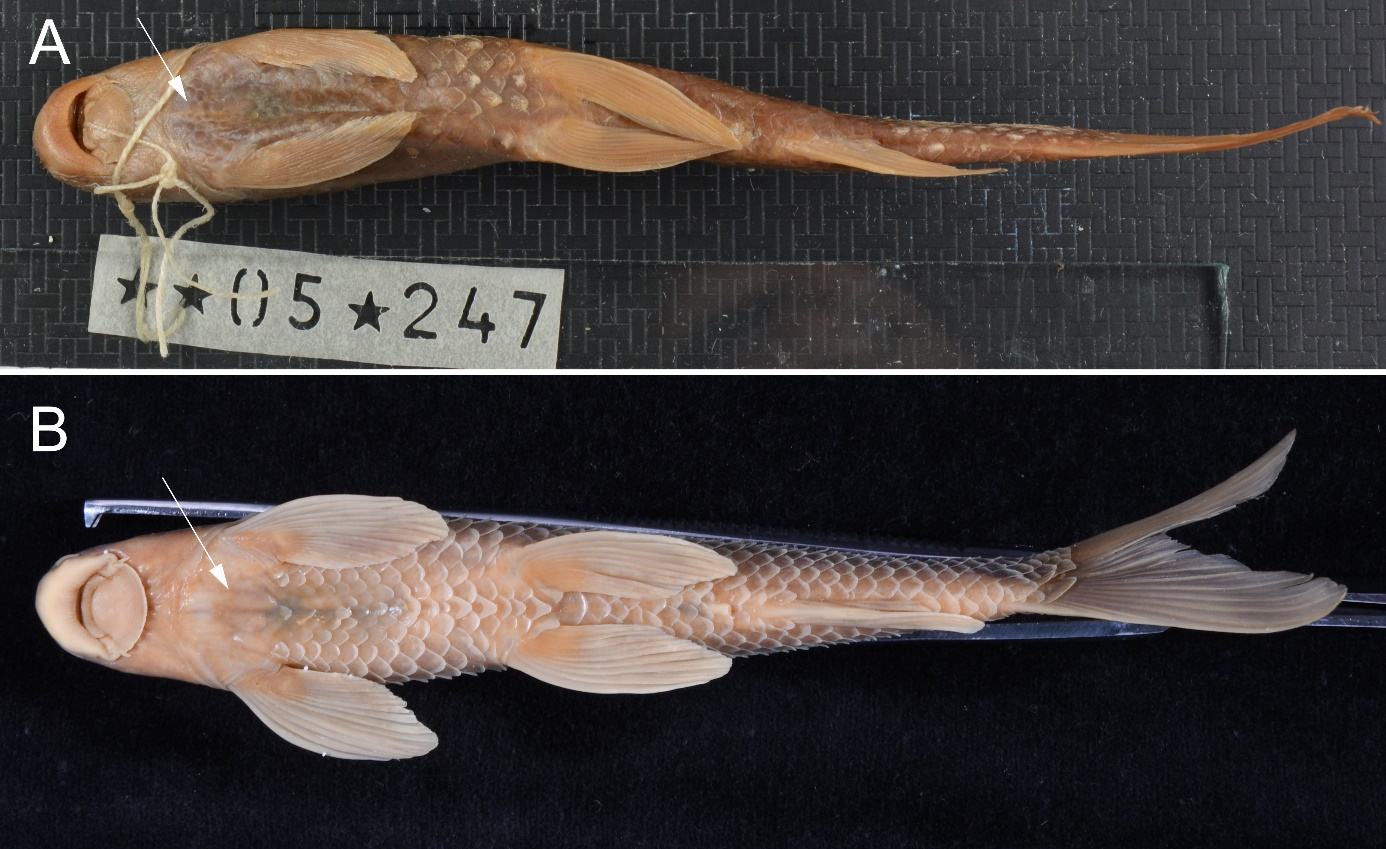


**Figure S3.** Ventral side of *Garra makiensis* from the Gotta River. **A** MNHN-1905-0247, syntype of *G. rothschildi*, 108.7 mm SL, The Muséum national d'Histoire naturelle, Paris; **B** AMNH 227323, Errer Gota [Gotta] River, Eastern side of Errer town, pools near main road, Hararge, Ethiopia (09°30'N, 41°15'E), The American Museum of Natural History. Arrows showing embedded scales on chest.


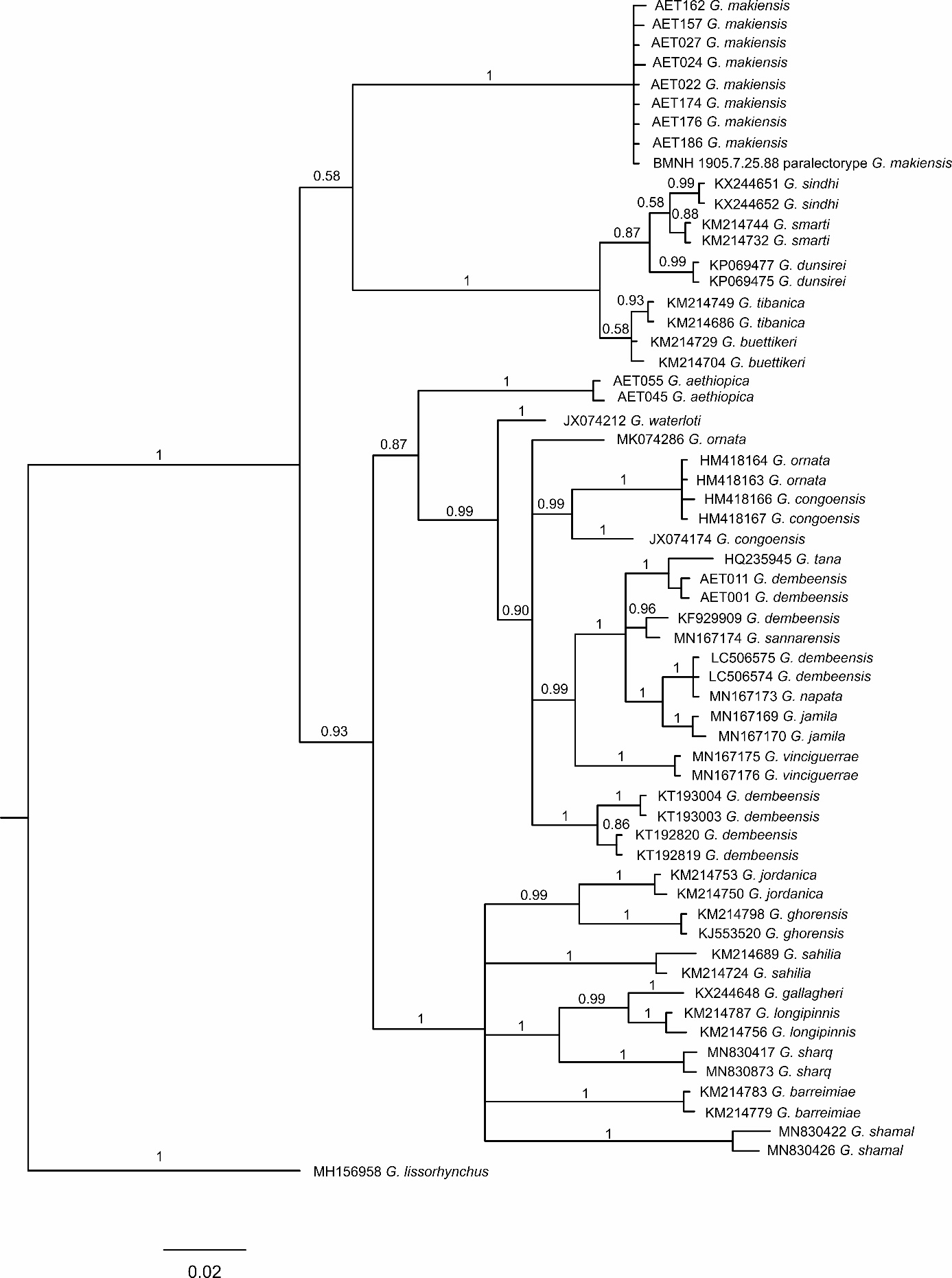


**Figure S4.** BI tree based on CO1 sequences.
